# Association between Serum Lactate and Morbidity and Mortality in Neonates: A Systematic Review and Meta-Analysis

**DOI:** 10.3390/children10111796

**Published:** 2023-11-08

**Authors:** Felipe Yu Matsushita, Vera Lucia Jornada Krebs, Werther Brunow De Carvalho

**Affiliations:** 1 Department of Pediatrics, Neonatology Division, Faculty of Medicine, University of São Paulo, São Paulo 01246-903, Brazil; vera.krebs@hc.fm.usp.br (V.L.J.K.); werther.brunow@hc.fm.usp.br (W.B.D.C.); 2 Instituto da Criança, Av. Dr. Enéas de Carvalho Aguiar, 647, São Paulo 05403-000, Brazil

**Keywords:** lactate, newborn, mortality, critical illness, preterm, neonates

## Abstract

Objective: Lactate is a marker of hypoperfusion in critically ill patients. Whether lactate is useful for identifying and stratifying neonates with a higher risk of adverse outcomes remains unknown. This study aimed to investigate the association between lactate and morbidity and mortality in neonates. Methods: A meta-analysis was performed to determine the association between blood lactate levels and outcomes in neonates. Ovid MEDLINE, EMBASE, Cochrane Library, and ClinicalTrials.gov were searched from inception to 1 May 2021. A total of 49 observational studies and 14 data accuracy test studies were included. The risk of bias was assessed using the Newcastle-Ottawa Scale for observational studies and the QUADAS-2 tool for data accuracy test studies. The primary outcome was mortality, while the secondary outcomes included acute kidney injury, necessity for renal replacement therapy, neurological outcomes, respiratory morbidities, hemodynamic instability, and retinopathy of prematurity. Results: Of the 3184 articles screened, 63 studies fulfilled all eligibility criteria, comprising 46,069 neonates. Higher lactate levels are associated with mortality (standard mean difference, −1.09 [95% CI, −1.46 to −0.73]). Using the estimated sensitivity (0.769) and specificity (0.791) and assuming a prevalence of 15% for adverse outcomes (median of prevalence among studies) in a hypothetical cohort of 10,000 neonates, assessing the lactate level alone would miss 346 (3.46%) cases (false negative) and wrongly diagnose 1776 (17.76%) cases (false positive). Conclusions: Higher lactate levels are associated with a greater risk of mortality and morbidities in neonates. However, our results do not support the use of lactate as a screening test to identify adverse outcomes in newborns. Research efforts should focus on analyzing serial lactate measurements, rather than a single measurement.

## 1. Introduction

Lactate is a powerful parameter that can be used to indirectly assess the hemodynamic system, but only when used correctly [1]. In critically ill patients, lactate is a classical marker, where its elevation is associated with greater morbidity and mortality [1]. Hyperlactatemia is a hallmark parameter in shock states because of lactate production in anaerobic metabolism, representing a state where there is an inadequate oxygen supply [2]. In adult and pediatric literature, there is strong evidence that lactate is a predictor of mortality [3,4]. Unfortunately, evidence about the utility of lactate measurement in neonates is limited.

There is no consensus regarding the treatment of hemodynamic instability in neonates, especially in preterm infants [5]. Moreover, classical parameters that are used to evaluate the cardiovascular system such as blood pressure alone are still not reliable in the neonatal period. Currently, there still is no definition for hypotension in neonates, nor a consensus of whether its correction is beneficial [6]. 

In this context, being able to use a parameter that aids in the diagnosis and treatment of hemodynamic instability in newborns would be valuable. The aim of this systematic review and meta-analysis was to determine the association between blood lactate concentration and morbidity and mortality in neonates.

## 2. Material and Methods

**Figure 1:**
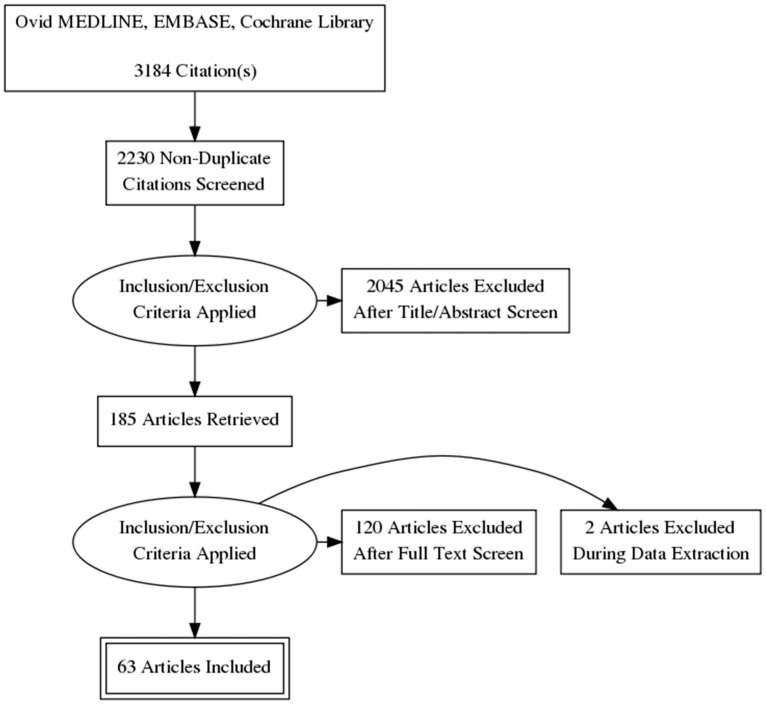
Flowchart—Preferred Reporting Items for Systematic Reviews and Meta-Analysis (PRISMA).

This systematic review and meta-analysis followed the recommendations based on the PRISMA (Preferred Reporting Items for Systematic Reviews and Meta-analysis) [7] and the Cochrane Centre for Reviews and Dissemination [8]. The search strategy was developed according to recommendations of PRESS [9] (Peer Review of Electronic Search Strategies) and was executed in May 2021. Ovid MEDLINE, EMBASE, Cochrane Library, and trial registries were searched without publication or language restrictions (see the Search Strategy in Figure S1). All references from retrieved citations were searched for additional relevant studies. The Rayyan web app [10] was used for study selection and initial abstract and title screening. The PRISMA flowchart is presented in Figure 1. We did not find any randomized controlled trials. Data extraction was performed by two authors (FYM and VLJK) and plotted in a previously built structured data extraction form. Any unresolved discrepancies of extracted data were resolved by a third author (WBC).

Two authors (FYM and VLJK) independently screened titles and abstracts and reviewed them. When the title and abstract were insufficient to decide on eligibility criteria, the full text was retrieved. If there was an unresolved disagreement, a third author (WBC) was consulted. All selected studies were retrieved and applied to a predefined inclusion criterion. The eligibility criteria included: (1) the study covered a neonatal population or a specific neonatal subgroup analyzed separately (<6 weeks postnatal age or < corrected gestational age of 40 weeks); (2) the study had at least one lactate measurement with a defined time assessment point; and (3) the study reported at least one outcome of interest. Studies that included pediatric patients were only eligible if data for neonates could be extracted separately. Studies reported only as abstracts were eligible only if sufficient information was available. If multiple articles analyzed the same set of patients, we included only the article with the largest number of neonates. This systematic review and meta-analysis followed the previously published protocol registered with the PROSPERO International Prospective Register of Systematic Reviews (CRD42021253329). Protocol changes are given in the Supplementary Material.

The primary objective was to evaluate the impact of hyperlactatemia on mortality in neonates during hospital stay. Composite outcomes with survival data were analyzed as mortality. Secondary outcomes included acute kidney injury, renal replacement therapy necessity, neurological outcomes, respiratory morbidities, hemodynamic instability, and retinopathy of prematurity.

We grouped the timing of lactate assessment into two different groups: early (lactate measured within 3 days of life or less) and late (lactate measured after more than 3 days of life). Lactate collected from the umbilical cord was analyzed separately. Initially, we planned to divide the lactate collected from venous and arterial sources, but due to insufficient data from the studies, this division was not possible. If studies assessed lactate at multiple time points, the earliest post-condition/intervention point or the highest value was selected. Hyperlactatemia was defined according to each study definition.

We assessed the risk of bias of included studies using the Newcastle-Ottawa Scale [11] for nonrandomized studies. A study with a total score of 7 or higher was considered of good quality, a study with a score of 4 to 6 was considered of fair quality, and a study with a score of lower than 4 was considered of poor quality. To assess the risk of bias of diagnostic accuracy studies, we used the QUADAS-2 tool [12]. 

For dichotomous variables, we used the odds ratio (OR) as the common measure of association with its respective 95% confidence interval. Lactate as a continuous variable was reported as standard mean differences (SMDs) with their respective 95% confidence interval. When studies reported medians and interquartile intervals, we used Wan et al.’s formula to infer the mean value and standard deviation [13]. To meta-analyze, we used random-effects models as proposed by Der Simonian and Laird because of the anticipated heterogeneity between studies. Statistical analysis was performed using RevMan 5 (Review Manager 5) software, v5.4, The Cochrane Collaboration. Heterogeneity was analyzed by performing subgroup analysis based on subgroup population and was measured using I^2^ statistics where estimates higher than 50% were considered as indicating significant heterogeneity. A *p*-value lower than 0.05 was considered statistically significant. 

Following the recommendations from the Cochrane Screening and Diagnostic Test Methods Group, one author (FYM) extracted diagnostic data and derived the number of true-positive, false-positive, true-negative, and false-negative cases. A second author (VLJK) checked the extracted data, and if a consensus was not reached, a third author (WBC) was consulted. We then created forest plots with 95% confidence intervals (Cis) for sensitivity and specificity using RevMan 5 (Review Manager 5) software, v5.4, The Cochrane Collaboration. A hierarchical summary ROC model was used because the reported cutoff levels for lactate differed among included studies. A meta-analysis of diagnostic test accuracy studies was performed using MetaDTA (web-based tool v2.0) [14], and estimates of sensitivity and specificity were calculated. Heterogeneity was assessed by analyzing the forest plots of sensitivity and specificity across studies. 

## 3. Results

Among 3184 records, a total of 185 potentially relevant articles were screened and fully retrieved (Figure 1). Of those, 63 studies, including 46,069 newborns (sample sizes ranged from 16 to 21,182 neonates), met the full inclusion criteria (Table 1 and Table S1). No randomized controlled trials were found. Studies excluded from the systematic review and the justification for their exclusion are presented in Table S2. The majority of studies (57%) were conducted in North America and Europe, 20 were conducted in Asia-Oceania, 6 were conducted in Latin America, and 1 was conducted in Africa. All studies were published between 1994 and 2021, with most studies (46 of 63) published after 2010. Among the studies, 14 evaluated lactates in preterm infants, 13 evaluated lactates in neonates with infants with congenital heart disease (CHD), and 12 evaluated lactates in neonates with birth asphyxia. The mean lactate levels in the nonsurvivor group varied between 2.2 and 23.42 mmoL/L. After applying the Newcastle-Ottawa Scale, 36 studies were labeled as being of good quality and 13 as fair quality (Table S3). The main potential sources of bias were “Representativeness of cohort” and “Comparability”.

We identified 14 studies analyzing lactate in data accuracy tests, comprising 39,540 patients. The characteristics of the included studies are summarized in Table S1. The cut-off levels for lactate ranged from 2.5 to 9.95 mmoL/L. The main potential source of bias was “Patient Selection”.

### 3.1. Mortality

**Figure 2:**
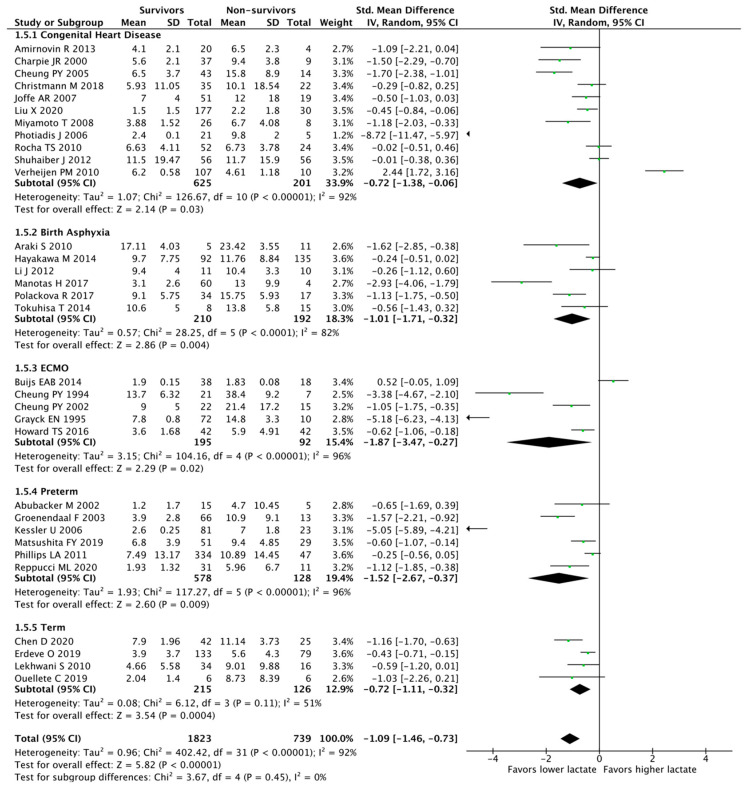
Meta-analysis of hyperlactatemia (continuous variable) and mortality stratified by neonatal population [15,16,17,18,19,20,21,22,23,24,25,26,27,28,29,30,31,32,33,34,35,36,37,38,39,40,41,42,43,44,45,46].

**Figure 3:**
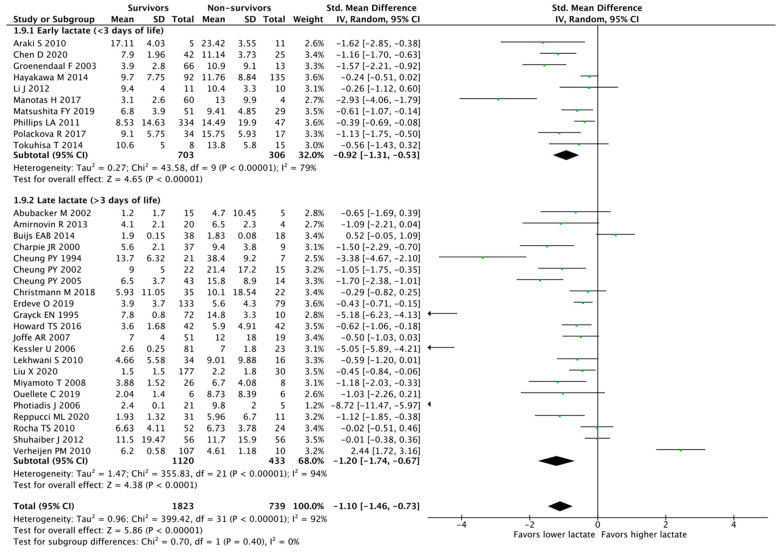
Meta-analysis of hyperlactatemia (continuous variable) and mortality stratified by time of lactate assessment (early vs. late) [15,16,17,18,19,20,21,22,23,24,25,26,27,28,29,30,31,32,33,34,35,36,37,38,39,40,41,42,43,44,45,46].

We found 32 studies analyzing hyperlactatemia as a continuous variable and mortality, comprising 2562 patients. Those who survived had lower lactate levels compared to nonsurvivors (SMD, −1.09 [95% CI, −1.46 to −0.73]; I^2^ = 92%; *p* < 0.00001). Eight studies evaluated mortality as part of the composite outcome. We grouped studies with similar neonatal populations, resulting in five subgroups: (1) congenital heart disease (SMD, −0.72 [95% CI, −1.38 to −0.06]; I^2^ = 92%; *n* = 826; *p* = 0.03); (2) birth asphyxia (SMD, −1.01 [95% CI, −1.71 to −0.32]; I^2^ 82%; *n* = 402; *p* = 0.004); (3) ECMO (SMD, −1.87 [95% CI, −3.47 to −0.27]; I^2^ = 96%; *n* = 287; *p* < 0.02); (4) preterm (SMD, −1.52 [95% CI, −2.67 to −0.73]; I^2^ 96%; *n* = 706; *p* = 0.009); and (5) term (SMD, −1.09 [95% CI, −1.11 to −0.32]; I^2^ = 51%; *n* = 341; *p* = 0.0004) (Figure 2). When dividing studies according to the time of lactate assessment, we categorized them into two different groups: (1) early (<3 days of life) (SMD, −0.92 [95% CI, −1.31 to −0.53], I^2^ = 79%; *n* = 1009; *p* < 0.00001) and (2) late (>3 days of life) (SMD, −1.2 [95% CI, −1.74 to −0.67], I^2^ = 94%; *n* = 1553; *p* < 0.00001) (Figure 3). The heterogeneity among studies was considerable (I^2^ = 92% for an overall impact of hyperlactatemia). 

**Figure 4:**
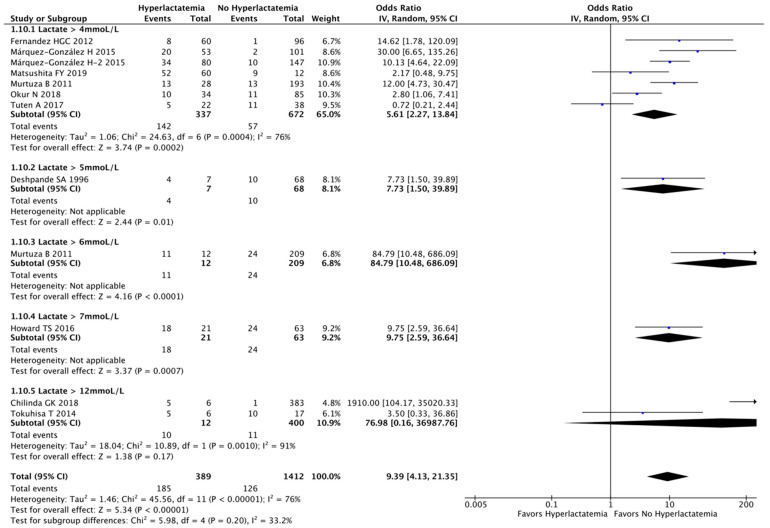
Meta-analysis of hyperlactatemia (dichotomous variable) and mortality stratified by hyperlactatemia definition [18,19,32,47,48,49,50,51,52,53].

We identified 12 studies evaluating hyperlactatemia as a dichotomous variable and its association with mortality, comprising 1801 patients. Hyperlactatemia was associated with a higher risk of mortality (OR, 9.39 [95% CI, 4.13–21.35]; I^2^ = 76%; *p* < 0.00001) (Figure 4). 

#### Adverse Outcomes

Hyperlactatemia is also associated with a higher risk of acute kidney injury (SMD, −0.68 [95% CI, −0.98 to −0.38]; I^2^ = 50%; *n* = 453; *p* < 0.00001), a higher risk of requiring renal replacement therapy in neonates with congenital heart disease (SMD, −0.84 [95% CI, −1.41 to −0.26]; I^2^ = 44%; *n* = 153; *p* = 0.004) (Figure S2), and worse neurological outcomes in neonates with birth asphyxia (SMD, -0.44 [95% CI, −0.67 to −0.22]; I^2^ = 0%; *n* = 307; *p* = 0.0001) (Figure S3).

Hyperlactatemia is not associated with a higher risk of respiratory morbidities, bronchopulmonary dysplasia (BPD), persistent ductus arteriosus (PDA), intraventricular hemorrhage (IVH), or retinopathy of prematurity (ROP) (Figures S4–S9).

However, higher lactate levels from umbilical cord blood are associated with a higher risk of worse outcomes (Figure S10).

### 3.2. Data Accuracy Test for Adverse Outcomes

**Figure 5:**
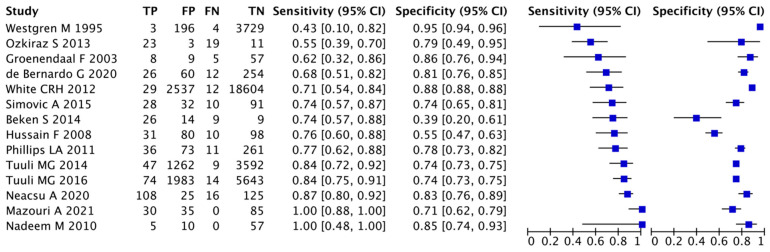
Forest plots of the sensitivity and specificity of lactate for adverse outcomes in neonates [33,36,55,56,59,63,64,65,66,67,68,69,70,71].

The estimate of sensitivity was 0.769 (95% CI, 0.692–0.831), and that of specificity was 0.791 (95% CI, 0.718–0.850). We observed a high heterogeneity among the studies, with a wide variety of sensitivity and specificity estimates. The prevalence rates of adverse outcomes ranged widely from 0.18% to 75%. We then applied the DTA estimates for sensitivity (0.769) and specificity (0.791) from our meta-analysis to a hypothetical cohort of 10,000 neonates with a prevalence rate of adverse outcomes of 0.18% (resulting in a median of 4.15 cases of adverse outcomes being missed and 2086 cases being wrongly diagnosed as an adverse outcome), 15% (resulting in a median of 346 cases of adverse outcomes being missed and 1776 being wrongly diagnosed as an adverse outcome), 50% (resulting in a median of 1155 cases of adverse outcomes being missed and 1045 cases being wrongly diagnosed as an adverse outcome), and 75% (resulting in a median of 1732 cases of adverse outcomes being missed and 522 being wrongly diagnosed as an adverse outcome). Summary receiver operating characteristic curves and forest plots are presented in Figure S11 and Figure 5, respectively. We applied the QUADAS-2 tool to assess the quality of studies, and the risk of bias was low (Figure S12).

## 4. Discussion

This systematic review and meta-analysis support the hypothesis that higher lactate levels are associated with increased mortality and risk of morbidities (AKI, RRT necessity, respiratory complications, hemodynamic instability, and neurological deficit) in neonates. This observation was similar across different subgroups of patients, from preterm infants to neonates with birth asphyxia. Although the data are robust and consistent, their interpretation is complicated due to the heterogeneity between studies, with different conditions and times of assessment. Indeed, the included studies had heterogeneity as high as 95%, even after subgrouping into more homogeneous groups, explaining the wide range of sensitivity (43–100%) and specificity (39–95%) in the data accuracy test for adverse outcomes. Using the estimated sensitivity (0.769) and specificity (0.791) and assuming a prevalence of 15% for adverse outcomes (the median of prevalence among studies) in a hypothetical cohort of 10,000 neonates, assessing lactate level alone would miss 346 cases (false negative) and wrongly diagnose 1776 cases (false positive).

Lactate is a widely used marker of altered tissue perfusion in critically ill patients, especially in adults, where hyperlactatemia is an indispensable feature that can be used to evaluate shock state. However, altered blood lactate cannot be attributed exclusively to anaerobic metabolism [72]. Other physiopathology mechanisms, including glycolysis, catecholamines release, liver hypoperfusion, and alterations in pyruvate dehydronegase activity (through mitochondrial dysfunction [73]) can contribute to an elevated lactate concentration [74]. Consequently, trying to define a cut-off for hyperlactatemia is difficult, unless the clinical condition and time of assessment are well determined. For instance, our meta-analysis showed that a lactate level greater than 4 mmoL/L was associated with higher mortality (OR, 5.61 [95% CI, 2.27–13.84]; I^2^ = 76%; *n* = 1009; *p* = 0.0002). However, when analyzing lactate as a continuous variable, we found 20 studies where the survivor group had a mean lactate level greater than 4 mmoL/L. Still, an elevated lactate level should always be a warning signal that requires evaluation [1]. Jansen TC et al. demonstrated that in adults, with increasing initial lactate levels, survival quickly decreased [75].

Therefore, without a clear neonatal subpopulation, clinical condition, and time of assessment, the lactate level alone is unlikely to assist as a screening test for adverse outcomes in newborns. However, as the neonates with the highest risk of death were those with a higher lactate concentration, lactate levels could be used to stratify those with a higher risk of adverse outcomes. These interpretations are in agreement with pediatric studies. Scott HF et al. [4], found that in children attending emergency departments, hyperlactatemia is associated with mortality, but with low sensitivity (20%). That is, lactate levels alone are not effective as a screening test, but might be used to identify the patients at highest risk. The evidence that lactate is a marker of severity of illness in adults is vast [73]. In fact, the SEPSIS-3 consensus requires a persistence of lactate greater than 2 mmoL/L to identify adult patients with sepsis with a greater risk of mortality [76]. For this reason, recent studies in adult and pediatric populations have focused on lactate clearance as the predictor of outcome, rather than the isolated lactate level itself [74,77,78,79]. In a recent systematic review evaluating adult patients, Jean-Louis Vincent et al. found that serial lactate measurement could be useful in the evaluation of the response to therapy in critically ill patients and stated that lactate clearance evaluation seems to be valid regardless of the initial value [74]. Despite the complexity of the interpretation of lactate level, its decrease is ultimately a good sign [80].

Future studies evaluating lactate levels in neonates need to adjust for potential confounders in lactate metabolism. For example, we did not find any study evaluating a possible interference of vasoactive drugs in lactate metabolism. It is known that the use of exogenous catecholamines induces an increased plasma lactate concentration [81]. Moreover, with recently published guidelines using point-of-care ultrasound to assess the hemodynamic state in neonates [82], lactate could be an additional parameter in conjunction with an echocardiogram.

Our systematic review and meta-analysis were conducted through a rigorous search strategy through all of the available literature, including four studies not written in English, with strong statistical analysis, and risk of bias assessment. However, several limitations are worthy of note. First, a meta-analysis of observational studies does not permit conclusions about causality. Second, we found a wide heterogeneity between studies, with varied subpopulations, clinical conditions, and lack of adjustment for covariates. This heterogeneity poses a challenge in determining whether the outcomes and studies are comparable or not. With our results, we suggest that further research evaluating blood lactate levels carefully adjust for potential confounders, including exogenous catecholamines administration. Moreover, research efforts should focus on inspecting serial lactate measurements, rather than a single measurement. Third, as there is no definition of hyperlactatemia in neonates, we found no study where the threshold was predefined in the data accuracy test analysis. Lastly, 13 of the included studies had a fair quality classification through the Newcastle-Ottawa Scale.

## 5. Conclusions

Our systematic review and meta-analysis, which included data from 46,069 neonates, suggest that greater lactate levels are associated with a higher risk of mortality and morbidities. Nonetheless, until new studies assess the precise clinical condition and time of assessment, the results from our meta-analysis do not support the use of lactate levels as a screening test to identify adverse outcome in newborns. Research efforts should focus on analyzing serial lactate measurements, rather than a single measurement.

## Figures and Tables

**Table 1:** Characteristics of the included studies in the systematic review.

Author	Year	Country	Study Type	Subgroup Population	No. Patients	Gestational Age	Birth Weight (kg)	Outcomes
Charpie JR [15]	2000	USA	PC	CHD	46	-	3.2 (0.5)	Death or ECMO
Polackova R [16]	2017	Czech republic	PC	Birth Asphyxia	51	38.8 (1.8)—adverse outcome group	3.2 (0.6)—adverse outcome group	Death or severe disability
Lekhwani S [17]	2010	India	RC	All	50	-	-	Death
Tokuhisa T [18]	2014	Japan	CC	Birth Asphyxia	23	38.5 (1.3)—adverse outcome group	2.9 (0.7)—adverse outcome group	Death or cerebral palsy
Matsushita FY [19]	2019	Brazil	RC	Preterm	80	26.1 (2.1)	0.66 (0.14)	Death
Buijs EAB [20]	2014	Netherlands	PC	ECMO	56	-	3 (2.2–3.3)	Death
Photiadis J [21]	2006	Germany	PC	CHD	26	-	3.3 (0.1)—nonsurvivor group	Death
Amirnovin R [22]	2013	USA	PC	CHD	24	-	3.3 (0.4)	Death OR surgical intervention OR ECMO OR transplant
Li J [23]	2012	Japan	RC	Birth Asphyxia	21	39.2 (1.9)—poor outcome group	2.8 (0.4)—poor outcome group	Death or neurological deficit
Shuhaiber J [24]	2012	USA	CC	CHD	112		25% with birth weight < 2.5 kg—nonsurvivor group	Death
Hayakawa M [25]	2014	Japan	RC	Birth Asphyxia	227	36.6 (38.4–40.6)	2.9 (2.6–3.2)	Death or neurological deficit
Joffe AR [26]	2007	Canada	PC	CHD	70	39 (2)	3.3 (0.6)	Death
Manotas H [27]	2017	Colombia	RC	Birth Asphyxia	64	-	-	Death
Liu X [28]	2020	China	RC	CHD	207	-	3 (0.5)—nonsurvivor group	Death
Ouellete C [29]	2019	USA	RC	Sepsis	12	-	-	Death
Miyamoto T [30]	2008	Germany	RC	CHD	34	35.5 (2.3)	2.1 (0.2)	Death
Rocha TS [31]	2010	Brazil	RC	CHD	76	-	3.1 (0.4)—nonsurvivor group	Death
Howard TS [32]	2016	USA	RC	CHD	84	-	2.9 (2.3–3.1)—nonsurvivor group	Death
Groenendaal F [33]	2003	Netherlands	RC	Preterm	79	28.5 (2.3)—poor outcome group	1.1 (0.5)—poor outcome group	Death or cerebral palsy
Christmann M [34]	2018	Switzerland	RC	CHD	57	-	2.9 (0.5)—nonsurvivor group	Death
Cheung PY [35]	1994	Canada	RC	ECMO	28	38.3 (2.1)—nonsurvivors	3 (0.4)—nonsurvivors	Death
Phillips LA [36]	2011	UK	PC	Preterm	381	28 (23–37)	1 (0.37–1.5)	Death
Kessler U [37]	2006	Switzerland	RC	Preterm/NEC	128	28.7 (0.8)—nonsurvivors	1.2 (0.12)—nonsurvivors	Death
Abubacker M [38]	2002	UK	RC	Preterm/NEC	24	27 (24–36)—nonsurvivors	0.7 (0.5–1.8)—nonsurvivors	Death
Verheijen PM [39]	2010	Netherlands	RC	CHD	105	-	-	Death
Araki S [40]	2010	Japan	RC	Birth Asphyxia	16	35.6 (4.5)—nonsurvivors	2.3 (0.7)—nonsurvivors	Death
Erdeve O [41]	2019	Turkey	PC	All	372	31.1 (5.4)—nonsurvivors	1.65 (1.09)—nonsurvivors	Death or ECMO
Chen D [42]	2020	China	PC	All	161	31.9 (3.5)—nonsurvivors	1.95 (0.53)—nonsurvivors	Death
Cheung PY [43]	2002	Canada	PC	ECMO	74	39 (2)	3.2 (0.7)	Death
Cheung PY [44]	2005	Canada	PC	CHD	85	38 (1)—nonsurvivors	3.1 (0.55)—nonsurvivors	Death
Reppucci ML [45]	2020	USA	RC	Preterm/GI perforation	42	-	BW < 1500 g	Death
Grayck EN [46]	1995	USA	RC	ECMO	82	-	-	Death/intracranial hemorrhage
Fernandez HGC [47]	2012	Brazil	RC	All	156	33.1 (4)—hyperlactatemia	1.83 (0.88)—hyperlactatemia	Death/seizure/pulmonary hypertension/intracerebral hemorrhage
Márquez-González H [48]	2015	Mexico	PC	All	154	18% > 37 weeks—nonsurvivors	22% > 2500 g—nonsurvivors	Death
Márquez-González-H [48]	2015	Mexico	PC	All	227	-	-	Death
Murtuza B [49]	2011	Switzerland	RC	CHD	221	-	3.1 (0.6)	Death
Okur N [50]	2018	Turkey	PC	Preterm	119	28.2 (2)—hyperlactatemia	0.96 (0.31)—hyperlactatemia	Death/MV duration/IVH/PDA/ROP/BPD
Tuten A [51]	2017	Turkey	PC	Preterm	60	27 (2.5)	0.99 (0.28)	Death/BPD/PDA/NEC/IVH/ROP
Deshpande SA [52]	1996	UK	PC	All mechanically ventilated	75	29 (23–40)	1.3 (0.55–4.08)	Death
Chilinda GK [53]	2018	Malawi	PC	All	389	-	2.9 (0.57)—hyperlactatemia	Death
Haiju Z [54]	2008	China	PC	Birth Asphyxia	18	38.1 (1.05)–moderate to severe HIE	2.7 (2.2–3.1)—moderate to severe HIE	Severe HIE
Neacsu A [55]	2020	Romania	RC	Birth Asphyxia	274	Term infants (>37 weeks)	-	APGAR < 3 first minute OR APGAR < 5 fifth minute OR respiratory insufficiency OR NICU > 24 h
Mazouri A [56]	2021	Iran	PC	Meconium Aspirate Syndrome	150	38.6 (1.43)	-	Pulmonary hemorrhage/pulmonary hypertension/IVH/MV necessity
Syed F [57]	2019	India	PC	Preterm	156	34–36 + 6/7 weeks	-	RDS/TTN/pneumonia/MAS
Karabayir N [58]	2014	Turkey	PC	All	1341	39.3 (0.9)	3.4 (0.6)	MAS/MV/O2 supply
Ozkiraz S [59]	2013	Turkey	CC	TTN	56	37.7 (1.6)	2.9 (0.5)	Respiratory support
Simovic AM [60]	2016	Serbia	CC	Preterm	108	31.7 (3.3)—respiratory support	1.8 (0.7)	Respiratory support
Miletin J [61]	2008	Ireland	PC	Preterm	38	26.5 (24–29)—low SVC group	1.1 (0.5–1.44)—Low SVC group	Low SVC
Balushi AA [62]	2017	Canada	RC	Birth Asphyxia	190	39.2 (1.5)—hypotension group	3.4 (0.6)—Hypotension group	Hypotension/brain injury

PC: Prospective cohort; RC: retrospective cohort; CC: case–control; CHD: congenital heart disease; NEC: necrotizing enterocolitis; GI: gastrointestinal; MV: mechanical ventilation; IVH: intraventricular hemorrhage; PDA: persistent ductus arteriosus; ROP: retinopathy of prematurity; BPD: bronchopulmonary dysplasia; HIE: hypoxic-ischemic encephalopathy; NICU: neonatal intensive care unit; RDS: respiratory distress syndrome; TTN: transient tachypnea of the newborn; MAS: meconium aspirate syndrome; SVC: superior vena cava flow.

## Data Availability

Data are contained within the article and supplementary materials.

## Supplementary Materials

The following supporting information can be downloaded at: https://www.mdpi.com/article/10.3390/children10111796/s1, Figure S1: Search Strategy; Figure S2: Meta-analysis of hyperlactatemia (Continuous Exposure) and Acute Kidney Injury/RRT necessity; Figure S3: Meta-analysis of hyperlactatemia (Continuous Exposure) in the first 24 hours of life in neonates with birth asphyxia and Neurological Outcomes; Figure S4: Meta-analysis of hyperlactatemia (Continuous Exposure) and Respiratory morbidities; Figure S5: Meta-analysis of hyperlactatemia > 4 mmoL/L (Dichotomous Exposure) and risk of Bronchopulmonary Dysplasia; Figure S6: Meta-analysis of hyperlactatemia (Continuous Exposure) and Hemodynamic Instability; Figure S7: Meta-analysis of hyperlactatemia > 4 mmoL/L (Dichotomous Exposure) and risk of Persistent Ductus Arteriosus; Figure S8: Meta-analysis of hyperlactatemia > 4 mmoL/L (Dichotomous Exposure) and risk of Intraventricular Hemorrhage; Figure S9: Meta-analysis of hyperlactatemia > 4 mmoL/L (Dichotomous Exposure) and risk of Retinopathy of Prematurity (ROP); Figure S10: Meta-analysis of hyperlactatemia (Continuous Exposure) from umbilical cord and Adverse Outcomes; Figure S11: Summary Receiver Operating Characteristics (SROC) plot of lactate for adverse outcomes; Figure S12: Summary of Risk of Bias using QUADAS-2 tool; Table S1: Characteristics of the DTA included studies in the systematic review; Table S2: Studies excluded from the meta-analysis with reason; Table S3: Assessment of risk of bias (Newcastle-Ottawa Scale). References [83,84,85,86,87,88,89,90,91,92,93,94,95,96,97,98,99,100,101,102,103,104,105,106,107,108,109,110,111,112,113,114,115,116,117,118,119,120,121,122,123,124,125,126,127,128,129,130,131,132,133,134,135,136,137,138,139,140,141,142,143,144,145,146,147,148,149,150,151,152,153,154,155,156,157,158,159,160,161,162,163,164,165,166,167,168,169,170,171,172,173,174,175,176,177,178,179,180,181,182,183,184,185,186,187,188,189,190,191,192,193,194,195,196,197,198,199,200,201,202,203,204] are cited in the supplementary materials.

## Abbreviations

CHD	Congenital heart disease
CI	Confidence interval
NICU	Neonatal intensive care unit
OR	Odds ratio
RRT	Renal replacement therapy
SD	Standard deviation
SMD	Standard mean deviation
Wk	Weeks
