# Supplementary material for: Association between Serum Lactate and Morbidity and Mortality in Neonates: A Systematic Review and Meta-Analysis
Source: Children (Basel). 2023 Nov 8;10(11):1796. doi: 10.3390/children10111796 (PMC10670916; doi:10.3390/children10111796)
Supplement: Supplementary file 1 [file children-10-01796-s001.zip › children-2639422-supplementary.pdf]

Supplemental Material

This Supplemental Material presents additional information

Association between Serum Lactate and Morbidity and Mortality in Neonates: A Systematic Review and Meta-Analysis

Felipe Yu Matsushita [1,2,*], Vera Lucia Jornada Krebs [1,2] and Werther Brunow De Carvalho [1,2]

[1] Department of Pediatrics, Neonatology Division, Faculty of Medicine, University of São Paulo, São Paulo 01246-903, Brazil; vera.krebs@hc.fm.usp.br (V.L.J.K.); werther.brunow@hc.fm.usp.br (W.B.D.C.)
[2] Instituto da Criança, Av. Dr. Enéas de Carvalho Aguiar, 647, São Paulo 05403-000, Brazil
* Correspondence: felipe.matsushita@hc.fm.usp.br; Tel.: +55-(11)-981800848

Figure S1. Search Strategy

Search strategy for the systematic literature search used on PubMed, EMBASE, and Cochrane Library

Newborn* OR neonat* OR preterm* OR perinat* OR Infant, Newborn OR Infant, Newborn, Intensive Care OR Newborn Infant OR Infant, Low Birth Weight OR Infant, Very Low Birth Weight OR Infant, Premature OR Infant, Extremely Low Birth Weight OR Intensive Care, Neonatal OR Infant, Small for Gestational Age OR Infant, Newborn, Diseases

AND

lactate* OR lactic acidosis OR blood lactate level OR hyperlactatemia OR serum lactate OR Lactates OR Lactic Acid OR Acidosis, Lactic

AND

Mechanical Ventilation* OR Hemodynamic Instability OR Acute Renal Failure OR Acute Kidney Failure OR Respiratory Failure OR Mortality OR Shock OR Necrotizing Enterocolitis OR Retinopathy of Prematurity OR Bronchopulmonary Dysplasia OR Pneumothorax OR Patent Ductus Arteriosus OR Respiration, Artificial OR Sepsis OR Shock OR Acute Kidney Injury OR Renal Replacement Therapy OR Dialysis OR Respiratory Insufficiency OR Pulmonary Edema OR Cardiotonic Agents OR Necrotizing Enterocolitis OR Retinopathy of Prematurity OR Bronchopulmonary Dysplasia OR Pneumothorax OR Patent Ductus Arteriosus

Figure S2. Meta-analysis of hyperlactatemia (Continuous Exposure) and Acute Kidney Injury/RRT necessity

Study or Subgroup	No-AKI Mean	No-AKI SD	No-AKI Total	AKI Mean	AKI SD	AKI Total	Weight	Std. Mean Difference IV, Random, 95% CI
**1.1.1 Congenital Heart Disease**								
Hammer MJ 2018	2	0.9	36	3.7	1.9	35	20.8%	−1.14 [−1.64, −0.63]
**Subtotal (95% CI)**			**36**			**35**	**20.8%**	**−1.14 [−1.64, −0.63]**
Heterogeneity: Not applicable								
Test for overall effect: Z = 4.42 (P < 0.00001)								
**1.1.2 Birth Asphyxia**								
Bozkurt O 2019	6.03	8.25	117	10.23	12.44	49	30.8%	−0.43 [−0.77, −0.10]
Mok TYD 2020	2.03	1.44	43	4.72	5.17	23	19.8%	−0.82 [−1.34, −0.29]
**Subtotal (95% CI)**			**160**			**72**	**50.6%**	**−0.57 [−0.93, −0.21]**
Heterogeneity: $Tau^2 = 0.02$; $Chi^2 = 1.47$, df = 1 (P = 0.23); $I^2 = 32\%$								
Test for overall effect: Z = 3.08 (P = 0.002)								
**1.1.3 Preterm**								
Stojanovic V 2014	3.3	3.1	111	5.2	4.8	39	28.6%	−0.52 [−0.89, −0.15]
**Subtotal (95% CI)**			**111**			**39**	**28.6%**	**−0.52 [−0.89, −0.15]**
Heterogeneity: Not applicable								
Test for overall effect: Z = 2.77 (P = 0.006)								
**Total (95% CI)**			**307**			**146**	**100.0%**	**−0.68 [−0.98, −0.38]**
Heterogeneity: $Tau^2 = 0.05$; $Chi^2 = 6.02$, df = 3 (P = 0.11); $I^2 = 50\%$								
Test for overall effect: Z = 4.45 (P < 0.00001)								
Test for subgroup differences: $Chi^2 = 4.19$, df = 2 (P = 0.12), $I^2 = 52.3\%$								

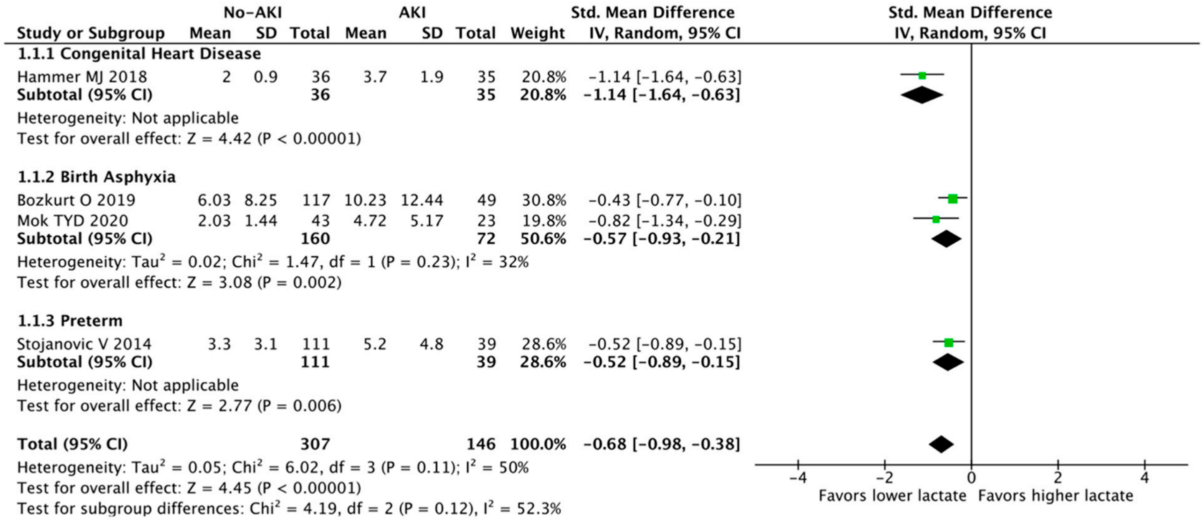

Study or Subgroup	No-RRT Mean	No-RRT SD	No-RRT Total	RRT Mean	RRT SD	RRT Total	Weight	Std. Mean Difference IV, Random, 95% CI
**1.2.1 Congenital Heart Disease**								
Hammer MJ 2018	1.8	0.8	63	3	1.9	8	36.7%	−1.22 [−1.99, −0.46]
Ozker E 2012	4.97	1.62	50	6.5	3.39	32	63.3%	−0.62 [−1.07, −0.16]
**Subtotal (95% CI)**			**113**			**40**	**100.0%**	**−0.84 [−1.41, −0.26]**
Heterogeneity: $Tau^2 = 0.08$; $Chi^2 = 1.80$, df = 1 (P = 0.18); $I^2 = 44\%$								
Test for overall effect: Z = 2.86 (P = 0.004)								
**Total (95% CI)**			**113**			**40**	**100.0%**	**−0.84 [−1.41, −0.26]**
Heterogeneity: $Tau^2 = 0.08$; $Chi^2 = 1.80$, df = 1 (P = 0.18); $I^2 = 44\%$								
Test for overall effect: Z = 2.86 (P = 0.004)								
Test for subgroup differences: Not applicable								

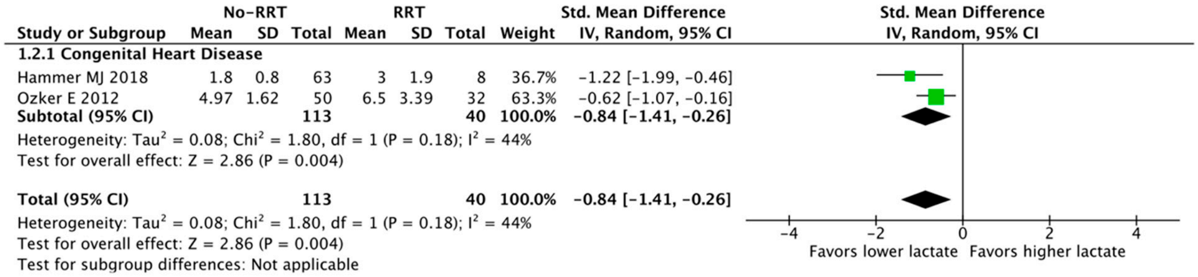

Figure S3. Meta-analysis of hyperlactatemia (Continuous Exposure) in the first 24 hours of life in neonates with birth asphyxia and Neurological Outcomes

Study or Subgroup	Better Outcome Mean	Better Outcome SD	Better Outcome Total	Worse Outcome Mean	Worse Outcome SD	Worse Outcome Total	Weight	Std. Mean Difference IV, Random, 95% CI
Balushi AA 2017	8.18	4.82	88	10.89	5.9	92	58.7%	−0.50 [−0.80, −0.20]
Beken S 2014	7.16	10.99	29	37.63	77.5	29	18.8%	−0.54 [−1.07, −0.02]
Chiang MC 2014	15.81	14.45	4	17.97	27.94	8	3.6%	−0.08 [−1.28, 1.12]
Michniewicz B 2020	8.12	14.08	27	11.68	14.83	30	19.0%	−0.24 [−0.76, 0.28]
**Total (95% CI)**			**148**			**159**	**100.0%**	**−0.44 [−0.67, −0.22]**
Heterogeneity: $Tau^2 = 0.00$; $Chi^2 = 1.20$, df = 3 (P = 0.75); $I^2 = 0\%$								
Test for overall effect: Z = 3.83 (P = 0.0001)								

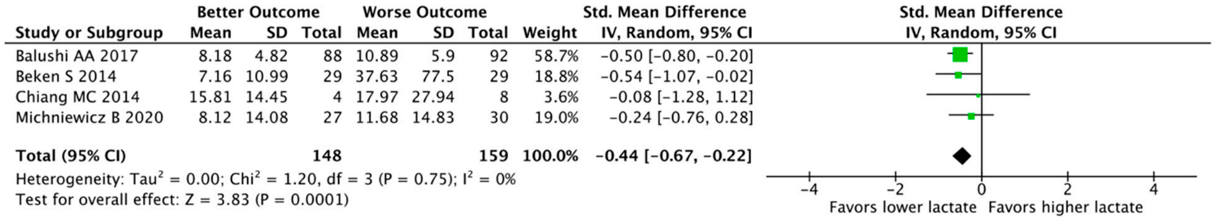

Figure S4. Meta-analysis of hyperlactatemia (Continuous Exposure) and Respiratory morbidities

	No-Respiratory Morbidity			Respiratory Morbidities				Std. Mean Difference
**Study or Subgroup**	**Mean**	**SD**	**Total**	**Mean**	**SD**	**Total**	**Weight**	**IV, Random, 95% CI**
**1.3.1 Term**								
Karabayir N 2014	4.9	3	1311	7.7	4.1	30	21.1%	−0.92 [−1.29, −0.56]
Mazouri A 2021	3.5	1	125	5.23	0.37	25	20.2%	−1.86 [−2.34, −1.38]
Ozkiraz S 2013	2.7	4.53	14	3.83	6.06	42	19.1%	−0.19 [−0.80, 0.41]
**Subtotal (95% CI)**			**1450**			**97**	**60.4%**	**−1.01 [−1.85, −0.16]**
Heterogeneity: $Tau^2 = 0.49$; $Chi^2 = 18.97$, df = 2 ($P < 0.0001$); $I^2 = 89\%$								
Test for overall effect: Z = 2.34 (P = 0.02)								
**1.3.2 Preterm**								
Simovic AM 2016	1.03	0.36	38	6.392	3.017	70	20.1%	−2.18 [−2.67, −1.69]
Syed F 2019	2.86	1.12	128	3.66	3.29	14	19.6%	−0.54 [−1.10, 0.01]
**Subtotal (95% CI)**			**166**			**84**	**39.6%**	**−1.37 [−2.97, 0.24]**
Heterogeneity: $Tau^2 = 1.27$; $Chi^2 = 18.61$, df = 1 ($P < 0.0001$); $I^2 = 95\%$								
Test for overall effect: Z = 1.67 (P = 0.09)								
**Total (95% CI)**			**1616**			**181**	**100.0%**	**−1.15 [−1.84, −0.46]**
Heterogeneity: $Tau^2 = 0.56$; $Chi^2 = 40.46$, df = 4 ($P < 0.00001$); $I^2 = 90\%$								
Test for overall effect: Z = 3.25 (P = 0.001)								
Test for subgroup differences: $Chi^2 = 0.15$, df = 1 (P = 0.70), $I^2 = 0\%$								

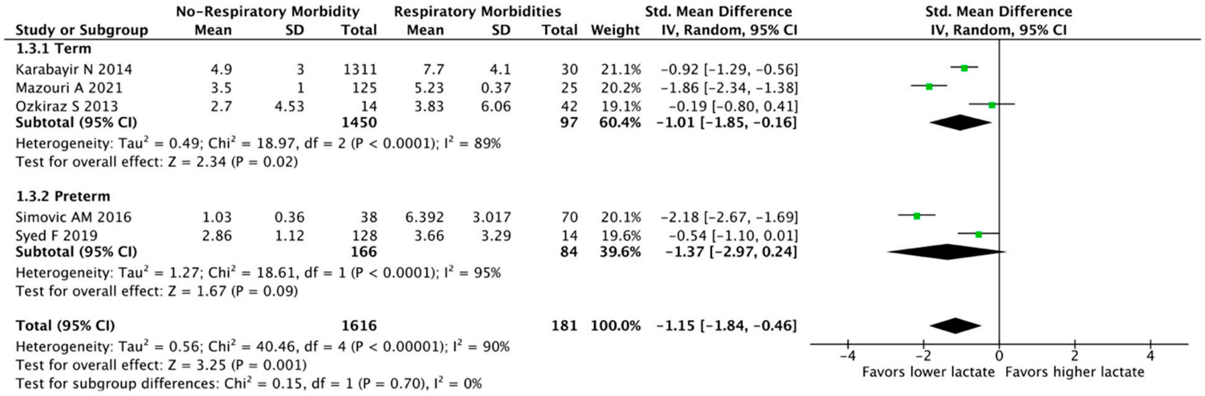

Figure S5. Meta-analysis of hyperlactatemia > 4mmoL/L (Dichotomous Exposure) and risk of Bronchopulmonary Dysplasia

	Hyperlactatemia		No Hyperlactatemia			Odds Ratio
**Study or Subgroup**	**Events**	**Total**	**Events**	**Total**	**Weight**	**M-H, Random, 95% CI**
Okur N 2018	4	34	15	85	46.7%	0.62 [0.19, 2.03]
Tuten A 2017	7	22	15	38	53.3%	0.72 [0.24, 2.17]
**Total (95% CI)**		**56**		**123**	**100.0%**	**0.67 [0.30, 1.50]**
Total events	11		30			
Heterogeneity: $Tau^2 = 0.00$; $Chi^2 = 0.03$, df = 1 (P = 0.87); $I^2 = 0\%$						
Test for overall effect: Z = 0.97 (P = 0.33)						

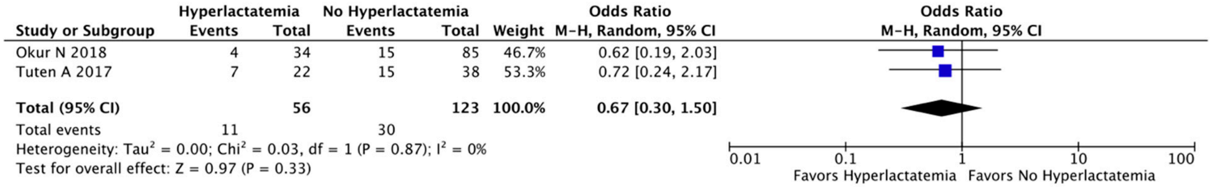

Figure S6. Meta-analysis of hyperlactatemia (Continuous Exposure) and Hemodynamic Instability

	Hemodynamic Stability			Hemodynamic Instability				Std. Mean Difference
**Study or Subgroup**	**Mean**	**SD**	**Total**	**Mean**	**SD**	**Total**	**Weight**	**IV, Random, 95% CI**
**1.4.1 Preterm**								
Miletin J 2008	3.6	4.43	30	4.93	5.09	8	18.2%	−0.29 [−1.07, 0.50]
**Subtotal (95% CI)**			**30**			**8**	**18.2%**	**−0.29 [−1.07, 0.50]**
Heterogeneity: Not applicable								
Test for overall effect: Z = 0.71 (P = 0.48)								
**1.4.2 Birth Asphyxia**								
Balushi AA 2017	7.13	3.96	36	10.92	6.24	154	81.8%	−0.64 [−1.01, −0.27]
**Subtotal (95% CI)**			**36**			**154**	**81.8%**	**−0.64 [−1.01, −0.27]**
Heterogeneity: Not applicable								
Test for overall effect: Z = 3.41 (P = 0.0006)								
**Total (95% CI)**			**66**			**162**	**100.0%**	**−0.58 [−0.91, −0.24]**
Heterogeneity: $Tau^2 = 0.00$; $Chi^2 = 0.65$, df = 1 (P = 0.42); $I^2 = 0\%$								
Test for overall effect: Z = 3.39 (P = 0.0007)								
Test for subgroup differences: $Chi^2 = 0.65$, df = 1 (P = 0.42), $I^2 = 0\%$								

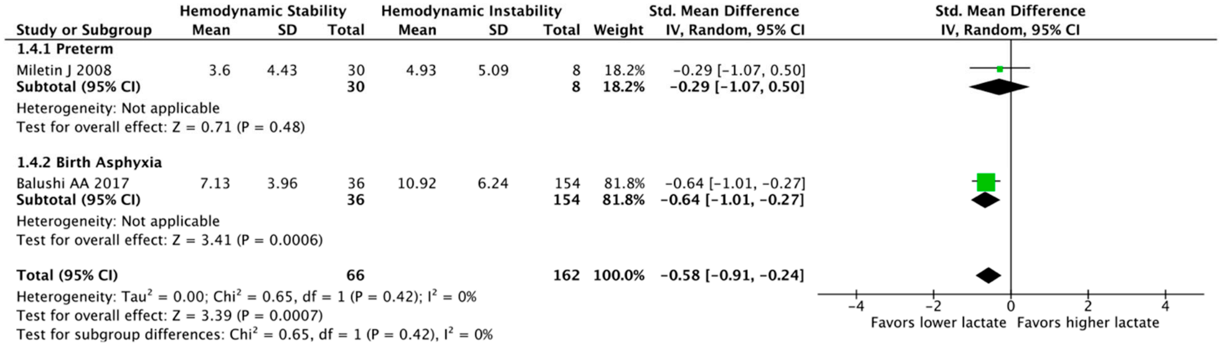

Figure S7. Meta-analysis of hyperlactatemia > 4mmoL/L (Dichotomous Exposure) and risk of Persistent Ductus Arteriosus

	Hyperlactatemia		No Hyperlactatemia			Odds Ratio
**Study or Subgroup**	**Events**	**Total**	**Events**	**Total**	**Weight**	**M-H, Random, 95% CI**
Okur N 2018	12	34	36	85	63.2%	0.74 [0.33, 1.69]
Tuten A 2017	8	22	16	38	36.8%	0.79 [0.27, 2.32]
**Total (95% CI)**		**56**		**123**	**100.0%**	**0.76 [0.39, 1.46]**
Total events	20		52			
Heterogeneity: $Tau^2 = 0.00$; $Chi^2 = 0.01$, df = 1 (P = 0.93); $I^2 = 0\%$						
Test for overall effect: Z = 0.83 (P = 0.41)						

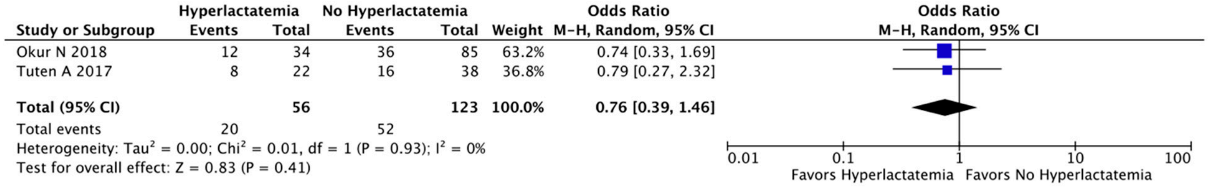

Figure S8. Meta-analysis of hyperlactatemia > 4mmoL/L (Dichotomous Exposure) and risk of Intraventricular Hemorrhage

	Hyperlactatemia		No Hyperlactatemia			Odds Ratio
Study or Subgroup	Events	Total	Events	Total	Weight	M-H, Random, 95% CI
Okur N 2018	5	34	5	85	48.3%	2.76 [0.74, 10.23]
Tuten A 2017	5	22	11	38	51.7%	0.72 [0.21, 2.44]
**Total (95% CI)**		**56**		**123**	**100.0%**	**1.38 [0.37, 5.14]**
Total events	10		16			

Heterogeneity: $Tau^2 = 0.48$; $Chi^2 = 2.16$, df = 1 (P = 0.14); $I^2 = 54\%$
Test for overall effect: Z = 0.48 (P = 0.63)

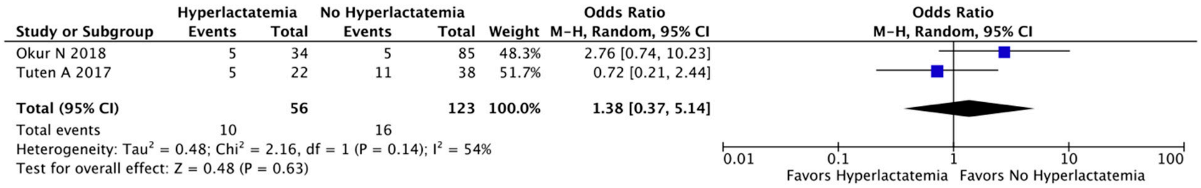

Figure S9. Meta-analysis of hyperlactatemia > 4mmoL/L (Dichotomous Exposure) and risk of Retinopathy of Prematurity (ROP).

	Hyperlactatemia		No Hyperlactatemia			Odds Ratio
Study or Subgroup	Events	Total	Events	Total	Weight	M-H, Random, 95% CI
Okur N 2018	2	34	9	85	51.2%	0.53 [0.11, 2.58]
Tuten A 2017	5	22	2	38	48.8%	5.29 [0.93, 30.11]
**Total (95% CI)**		**56**		**123**	**100.0%**	**1.62 [0.17, 15.59]**
Total events	7		11			

Heterogeneity: $Tau^2 = 1.94$; $Chi^2 = 3.69$, df = 1 (P = 0.05); $I^2 = 73\%$
Test for overall effect: Z = 0.42 (P = 0.67)

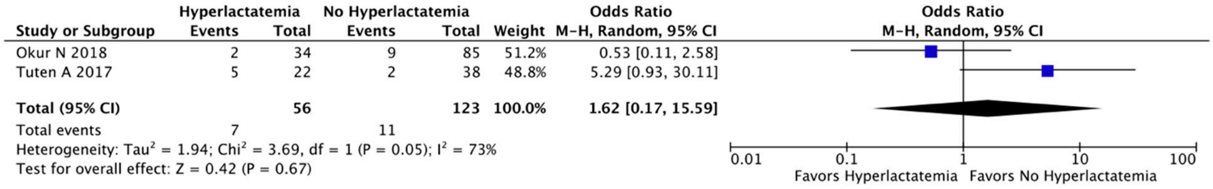

Figure S10. Meta-analysis of hyperlactatemia (Continuous Exposure) from umbilical cord and Adverse Outcomes

	Better Outcomes			Worse Outcomes				Std. Mean Difference
Study or Subgroup	Mean	SD	Total	Mean	SD	Total	Weight	IV, Random, 95% CI
**1.8.1 Birth Asphyxia**								
Haiju Z 2008	5.41	0.92	10	6.44	1.24	8	20.2%	-0.92 [-1.90, 0.07]
Neacsu A 2020	2.78	0.7	150	5.35	1.5	124	27.5%	-2.26 [-2.56, -1.96]
**Subtotal (95% CI)**			**160**			**132**	**47.7%**	**-1.67 [-2.98, -0.37]**
Heterogeneity: $Tau^2 = 0.76$; $Chi^2 = 6.48$, df = 1 (P = 0.01); $I^2 = 85\%$								
Test for overall effect: Z = 2.51 (P = 0.01)								
**1.8.2 Other**								
Mazouri A 2021	3.25	0.94	120	5.25	0.42	30	26.1%	-2.31 [-2.78, -1.83]
Syed F 2019	2.86	1.04	134	4.16	3.56	22	26.2%	-0.79 [-1.25, -0.33]
**Subtotal (95% CI)**			**254**			**52**	**52.3%**	**-1.55 [-3.03, -0.07]**
Heterogeneity: $Tau^2 = 1.09$; $Chi^2 = 19.92$, df = 1 (P < 0.00001); $I^2 = 95\%$								
Test for overall effect: Z = 2.05 (P = 0.04)								
**Total (95% CI)**			**414**			**184**	**100.0%**	**-1.62 [-2.43, -0.80]**

Heterogeneity: $Tau^2 = 0.61$; $Chi^2 = 34.14$, df = 3 (P < 0.00001); $I^2 = 91\%$
Test for overall effect: Z = 3.87 (P = 0.0001)
Test for subgroup differences: $Chi^2 = 0.02$, df = 1 (P = 0.90), $I^2 = 0\%$

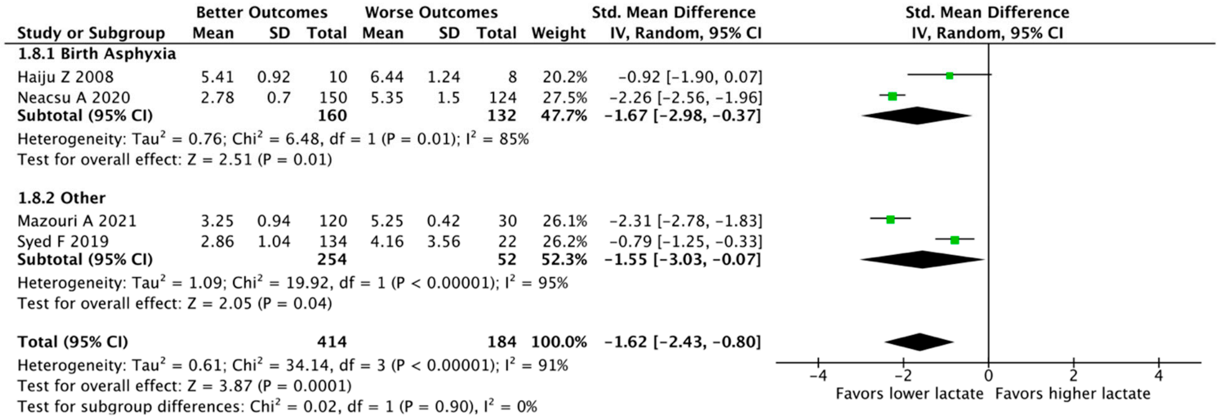

Figure S11. Summary Receiver Operating Characteristics (SROC) plot of lactate for adverse outcomes

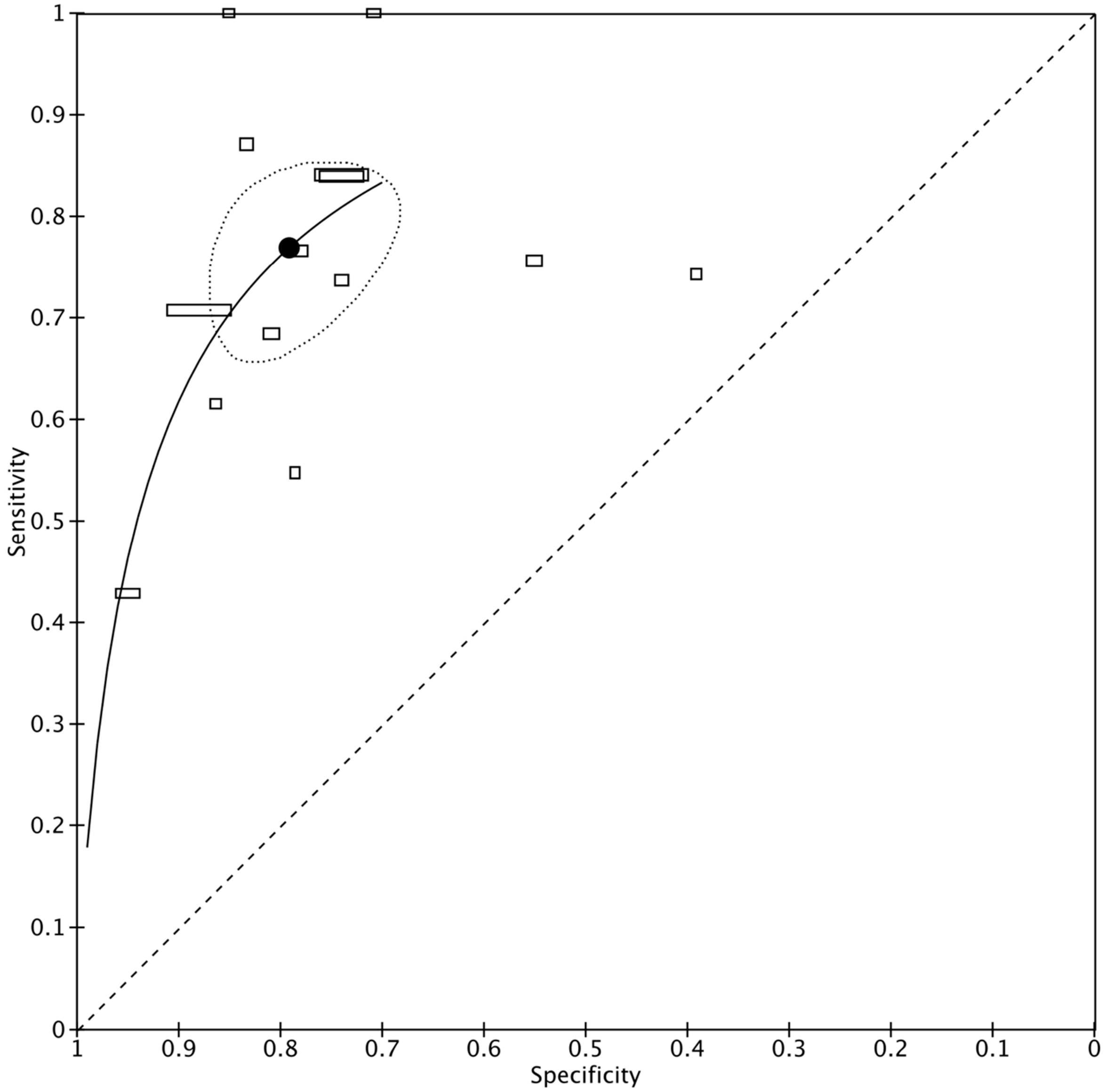

Figure S12. Summary of Risk of Bias using QUADAS-2 tool

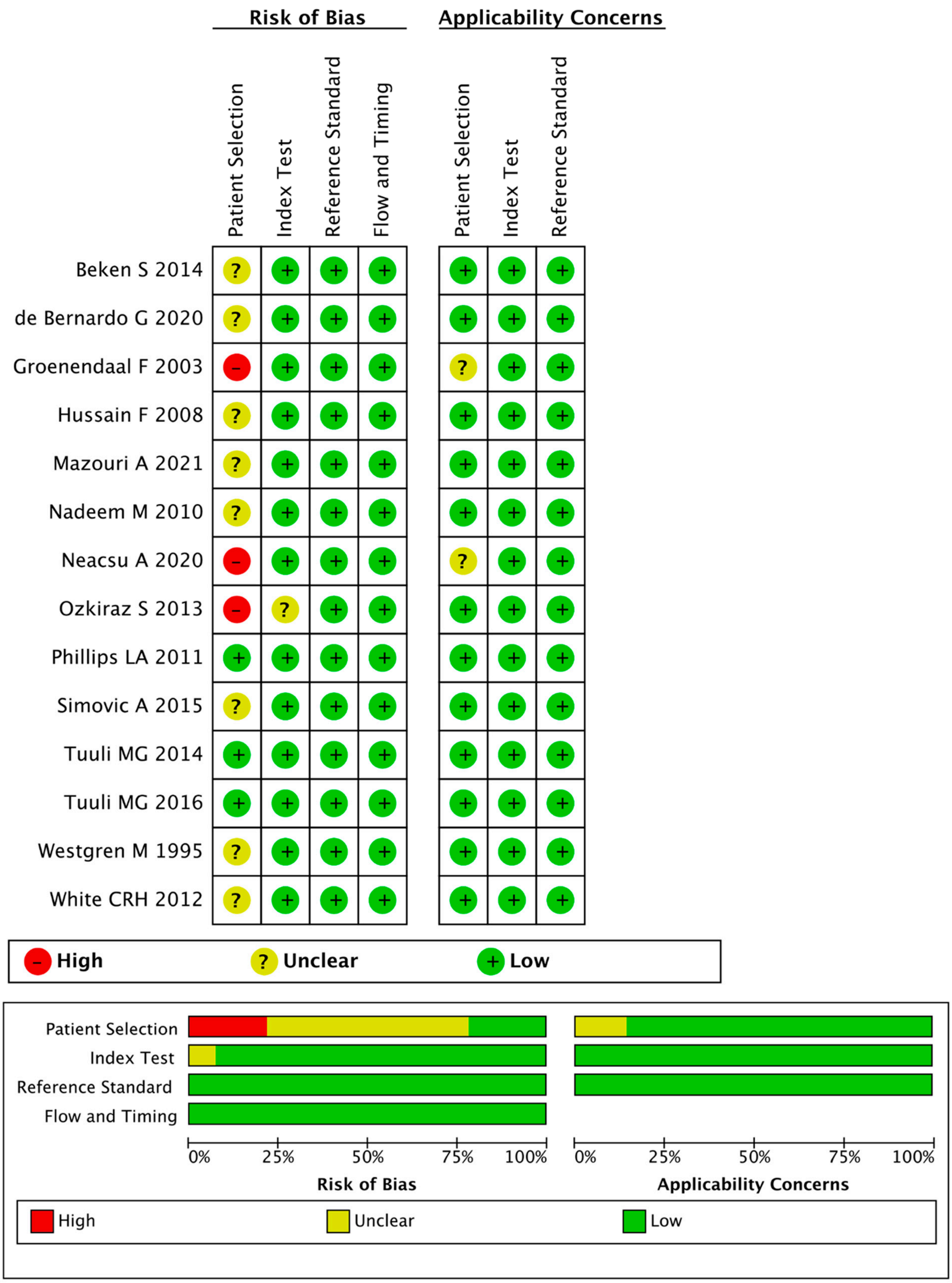

Changes between protocol and review

We included studies analyzing patients with less than 6 weeks postnatal age rather than the proposed 4 weeks postnatal age.

Meta-analysis of diagnostic test accuracy of lactate and adverse outcomes was added and QUADAS-2 tool was used to assess risk of bias

Table S1. Characteristics of the DTA included studies in the systematic review

Author	Country	Population	Cut-off level, mmoL/L	Adverse Outcome	Total	True Positive	False Positive	False Negative	True Negative	Sensitivity	Specificity
Neacsu A 2020 [55]	Romania	Birth Asphyxia	3.75	APGAR < 3 1o min OR < 5 5o min OR respiratory insufficiency OR NICU > 24h	274	108	25	16	125	0.87 (0.8 – 0.92)	0.83 (0.76 – 0.89)
White CRH 2012 [63]	Australia	Birth Asphyxia	5.7	Moderate-Severe HIE	21182	29	2537	12	18604	0.71 (0.54 – 0.84)	0.88 (0.88 – 0.88)
Tuuli MG 2014 [64]	USA	All	4	Death OR Endotracheal Intubation OR Mechanical Ventilation OR HIE OR MAS	4910	47	1262	9	3592	0.84 (0.72 – 0.92)	0.74 (0.73 – 0.75)
Mazouri A 2021 [56]	Iran	Meconium Aspirate Syndrome	4.1	Pulmonary Hemorrhage OR Pulmonary Hypertension OR Intraventricular Hemorrhage OR Mechanical Ventilation	150	30	35	0	85	1 (0.88 – 1)	0.71 (0.62 – 0.79)
Groenendaal F 2003 [33]	Netherlands	Preterm	5.7	Death OR Cerebral Palsy	79	8	9	5	57	0.62 (0.32 – 0.86)	0.86 (0.76 – 0.94)
Beken S 2014 [65]	Turkey	Birth Asphyxia	4.4	Stage III Sarnat	58	26	14	9	9	0.74 (0.57 – 0.88)	0.39 (0.2 – 0.61)
Nadeem M 2010 [66]	Ireland	Preterm	5.6	Death OR Severe IVH OR PVL	72	5	10	0	57	1 (0.48 – 1)	0.85 (0.74 – 0.93)
Phillips LA 2011 [36]	UK	Preterm	6.9	Death	381	36	73	11	261	0.77 (0.62 – 0.88)	0.78 (0.73 – 0.82)
Simovic A 2015 [67]	Serbia	All	9.95	Perinatal Asphyxia	161	28	32	10	91	0.74 (0.57 – 0.87)	0.74 (0.65 – 0.81)
De Bernardo G 2020 [68]	Italy	Full Term	8.25	NICU necessity	352	26	60	12	254	0.68 (0.51 – 0.82)	0.81 (0.76 – 0.85)
Ozkiraz S 2013 [59]	Turkey	Transitory Tachypnea of Newborn	2.5	Respiratory Support	56	23	3	19	11	0.55 (0.39 – 0.70)	0.79 (0.49 – 0.95)
Tuuli MG 2016 [69]	USA	Full Term	3.9	Death OR HIE OR EOT OR MV OR MAS	7714	74	1983	14	5643	0.84 (0.75 – 0.91)	0.74 (0.73 – 0.75)
Westgren M 1995 [70]	Sweden	All	3.72	Death	3932	3	196	4	3729	0.43 (0.10 – 0.82)	0.95 (0.94 – 0.96)
Hussain F 2009 [71]	Australia	Extremely Low Birth Weight	3.2	Death	219	31	80	10	98	0.76 (0.60 – 0.88)	0.55 (0.47 – 0.63)

Table S2 – Studies excluded from the meta-analysis with reason

Title	Author	Reason
Two-year survival and mental and psychomotor outcomes after the Norwood procedure: an analysis of the modified Blalock-Taussig shunt and right ventricle-to-pulmonary artery shunt surgical eras.[83]	Atallah, J et al.	Lactate analyzed as outcome
Outcomes of infants weighing three kilograms or less requiring extracorporeal membrane oxygenation after cardiac surgery.[84]	Bhat P et al.	Excluded patients < 30 days old
Impact of antenatal diagnosis of hypoplastic left heart syndrome on the clinical presentation and surgical outcomes: the Australian experience.[85]	Sivarajan V et al.	Lactate analyzed as outcome
Association of Hematocrit and Red Blood Cell Transfusion with Outcomes in Infants Undergoing Norwood Operation.[86]	Gupta P et al.	Lactate analyzed as outcome
Early lactate elevations following resuscitation from pediatric cardiac arrest are associated with increased mortality[87]	Topjian AA et al.	No specific newborn group
Intraoperative lactate levels and postoperative complications of pediatric cardiac surgery.[88]	Alves RL et al.	No specific newborn group
The use of arginine vasopressin in postoperative norwood patients[89]	Burton GL et al.	Lactate analyzed as outcome
Hyperlactatemia in neonates admitted to the cardiac intensive care unit with critical heart disease.[90]	Rossi AF et al.	Not enough data related to mortality group
Lactat acidosis – marker of severity of perinatal hypoxia [91]	Kuzovlev A et al.	Abstract. No outcome of interest
Early-onset thrombocytopenia in near-term and term infants with perinatal asphyxia[92]	Boutaybi N et al.	Lactate analyzed as outcome
Role of umbilical cord arterial pH and lactate in newborn assessment of term antenatal women with hypertensive disorders of pregnancy[93]	Kumar N et al.	Lactate dehydrogenase
Acute kidney injury assessed by prifle score and its relationship with metabolic markers and outcome[94]	Freire KMS et al.	Neonates were excluded
Randomized comparison between mild and moderate hypothermic cardiopulmonary bypass for neonatal arterial switch operation[95]	Aydemir NA et al.	Lactate as analyzed outcome
Association of lactate/albumin ratio level to organ failure and mortality in severe sepsis in a pediatric intensive care unit in Egypt[96]	Moustafa A et al.	No neonatal group
Hypothermia therapy after pediatric cardiac arrest[97]	Doherty D et al.	No neonatal group
Red Cell Transfusion Practices in Neonatal Intensive Care Unit: An Experience from Tertiary Care Centre[98]	Dogra K et al.	Lactate analyzed as outcome

Postoperative Abdominal NIRS Values Predict Low Cardiac Output Syndrome in Neonates[99]	Hickok RL et al.	Lactate as definition of low cardiac output syndrome
Early hyperlactataemia in critically ill children.[100]	Hatherill M et al.	No newborn group
Use of extracorporeal membrane oxygenation in postcardiotomy pediatric patients: parameters affecting survival[101]	Ergun S et al.	No newborn group
The strong ion gap predicts mortality in children following cardiopulmonary bypass surgery.[102]	Durward et al.	No specific newborn group
The utility of early lactate testing in undifferentiated pediatric systemic inflammatory response syndrome.[103]	Scott HF et al.	No specific newborn group
Therapeutic Plasma Exchange in Neonatal Septic Shock: A Retrospective Cohort Study[104]	Sawyer T et al.	Case Report
Lactate clearance prognosticates outcome in pediatric septic shock during first 24 h of intensive care unit admission.[105]	Nazir M et al.	No specific newborn group
Prophylactic milrinone for the prevention of low cardiac output syndrome and mortality in children undergoing surgery for congenital heart disease[106]	Burkhardt B et al.	Systematic Review
[Prognostic markers of mortality after congenital heart defect surgery].[107]	García-Hernández JA et al.	No specific newborn group
Clinical outcome score predicts the need for neurodevelopmental intervention after infant heart surgery.[108]	Mackie AS et al.	Time to lactate < 2.0
Functional Status of Neonatal and Pediatric Patients After Extracorporeal Membrane Oxygenation.[109]	Cashen K et al.	No specific newborn group
Blood lactate levels differ significantly between surviving and nonsurviving patients within the same risk-adjusted Classification for Congenital Heart Surgery (RACHS-1) group after pediatric cardiac surgery.[110]	Gonen MVY et al.	No specific newborn group
Lactic acidosis: A highly indicator of unfavorable outcome in critically ill children[111]	Neamtu ML et al.	No newborn group / Abstract
LOW-DOSE PROSTAGLANDIN E1 FOR CONGENITAL HEART DISEASE: IS IT TIME TO REVISIT THE DOSING GUIDELINES[112]	Vari D et al.	Lactate analyzed as outcome
Extracorporeal membrane oxygenator support in infants with systemic-pulmonary shunts.[113]	Botha P et al.	No specific newborn group
Post-cardiotomy extracorporeal cardiopulmonary resuscitation in neonates with complex single ventricle: analysis of outcomes.[114]	Polimenakos AC et al.	No specific newborn group
Central Venous to Arterial C o 2 Difference after Cardiac Surgery in Infants and Neonates∗[115]	Rhodes L et al.	No specific newborn group. < 90 days
[Prognostic factors associated with postoperative morbidity in children with isolated ventricular septal defect].[116]	Castro-Rodríguez CO et al.	No specific newborn group

Children undergoing heart transplant are at increased risk for postoperative vasodilatory shock[117]	Killinger JS et al.	No specific newborn group
Serial blood lactate levels as a predictor of mortality in children after cardiopulmonary bypass surgery.[118]	Kalyanaraman M et al.	No specific newborn group
Rate of increase in serum lactate level risk-stratifies infants after surgery for congenital heart disease.[119]	Schumacher KR et al.	No specific newborn group
Early prediction of capillary leak syndrome in infants after cardiopulmonary bypass.[120]	Kubicki R et al.	No specific newborn group
Survival and Mid-Term Neurologic Outcome after Extracorporeal Cardiopulmonary Resuscitation in Children[121]	Kramer P et al.	No specific newborn group
Cardiac troponin T levels for risk stratification in pediatric open heart surgery.[122]	Mildh LH et al.	No specific newborn group
Risk stratification of critically ill children and neonates with acute general surgical pathology requiring stabilisation, transfer to tertiary care facility and factors predicting mortality[123]	Kanaris C et al.	No specific newborn group
[Mortality-associated factors in pediatric patients with Blalock-Taussig shunt].[124]	Aguilar-Segura PR et al.	No specific newborn group
Acute kidney injury scoring system is a better predictor of increased length compared to inotrope score[125]	Olshove V et al.	Abstract - incomplete information - no age information - no lactate information
Risk factors for mechanical ventilation time after congenital heart surgery[126]	Manso P et al.	Abstract - incomplete information
Goal-directed medical therapy and point-of-care testing improve outcomes after congenital heart surgery.[127]	Rossi AF et al.	No specific newborn group
Prognostic value of perioperative near-infrared spectroscopy during neonatal and infant congenital heart surgery for adverse in-hospital clinical events.[128]	Dodge-Khatami J et al.	No specific newborn group
Initial postoperative serum lactate levels predict survival in children after open heart surgery.[129]	Siegel LB et al.	No specific newborn group
Moderate versus deep hypothermia for the arterial switch operation--experience with 100 consecutive patients.[130]	Rastan AJ et al.	Lactate analyzed as outcome
Levosimendan infusion in newborns after corrective surgery for congenital heart disease: randomized controlled trial[131]	Ricci Z et al.	Lactate analyzed outcome
Vasoactive-ventilation-renal score reliably predicts hospital length of stay after surgery for congenital heart disease[132]	Scherer B et al.	No specific newborn group
Predictors for an unsuccessful INtubation-SURfactant-Extubation procedure: a cohort study.[133]	Brix N et al.	Lactate analyzed as outcome
Venoarterial extracorporeal membrane oxygenation support for neonatal and pediatric refractory septic shock: more than 15 years of learning[134]	Solé A et al.	No specific newborn group

Prediction of pediatric sepsis mortality within 1 h of intensive care admission.[135]	Schlapbach LJ et al.	No specific newborn group
Changes in whole blood lactate levels during cardiopulmonary bypass for surgery for congenital cardiac disease: an early indicator of morbidity and mortality.[136]	Munoz R et al.	No specific newborn group
Prenatal Diagnosis of Transposition of the Great Arteries Reduces Postnatal Mortality: A Population-Based Study[137]	Nagata H et al.	Lactate analyzed as outcome. No outcome of interest
Utilisation of near infrared spectroscopy (NIRS) in monitoring haemodynamic stability of infants with hypoplastic left heart syndrome (HLHS) in the presurgical setting[138]	Woods P et al.	Lactate analyzed as outcome
Initial single-center experience with levosimendan infusion for perioperative management of univentricular heart with ductal-dependent systemic circulation.[139]	Garisto C et al.	Lactate analyzed as outcome
Prenatal detection of transposition of the great arteries does not reduce mortality and morbidity[140]	Jaeggi E et al.	Lactate analyzed as outcome
Extracorporeal membrane oxygenation for refractory septic shock in children: Our institution's results[141]	Rodríguez J et al.	No specific newborn group. Lactate as outcome
The relationship between blood lactate concentration, the Paediatric Index of Mortality 2 (PIM2) and mortality in paediatric intensive care.[142]	Morris KP et al.	No specific newborn group
Predictors of mortality at initiation of peritoneal dialysis in children after cardiac surgery.[143]	Boigner H et al.	No specific newborn group
Prenatal Diagnosis of Single Ventricle Physiology Impacts on Cardiac Morbidity and Mortality[144]	Weber RW et al.	Lactate analyzed as outcome
Risk factors and outcome of acute kidney injury after congenital heart surgery: A prospective observational study[145]	Amini S et al.	No specific newborn group
Relationship of serum procalcitonin, c-reactive protein, and lactic acid to organ failure and outcome in critically ill pediatric population[146]	Siddiqui I et al.	No specific newborn group
The impact of intraoperative vasopressin infusion in complex neonatal cardiac surgery[147]	Nishibe S et al.	Lactate analyzed as outcome
Beneficial effect of fenoldopam mesylate in preventing peak blood lactate level during cardiopulmonary bypass for paediatric cardiac surgery[148]	Ressia L et al.	No specific newborn group. Lactate as outcome
Incidence of milrinone blood levels outside the therapeutic range and their relevance in children after cardiac surgery for congenital heart disease.[149]	Guerra GG et al.	No specific newborn group
Correlation between cerebral-renal near-infrared spectroscopy and ipsilateral renal perfusion parameters as clinical outcome predictors after open heart surgery in neonates and infants.[150]	Ormeci T et al.	No specific newborn group. Lactate as outcome

Prospective validation of the vasoactive-inotropic score and correlation to short-term outcomes in neonates and infants after cardiothoracic surgery[151]	Davidson J et al.	No specific newborn group
Experience with extracorporeal life support in pediatric patients after cardiac surgery.[152]	Huang SC et al.	No specific newborn group
Perfusion index in the very preterm infant.[153]	Kinoshita M et al.	Lactate analyzed as outcome. No outcome of interest
Effect of Vasopressin on Systemic and Pulmonary Hemodynamics in Neonates[154]	Budniok T et al.	Lactate analyzed as outcome
Continuous renal replacement therapy in the NICU; Ten years' experience in a singlecenter[155]	Sawada M et al.	No specific newborn group. Lactate as outcome
Exchange transfusion in the treatment of neonatal septic shock: A ten-year experience in a neonatal intensive care unit[156]	Pugni L et al.	Lactate analyzed as outcome
Increasing duration of circulatory arrest, but not antegrade cerebral perfusion, prolongs postoperative recovery after neonatal cardiac surgery[157]	Algra S et al.	Lactate analyzed as outcome
Efficacy and safety of milrinone in preventing low cardiac output syndrome in infants and children after corrective surgery for congenital heart disease.[158]	Hoffman TM et al.	Lactate analyzed as outcome
Vasoactive inotropic score and outcome assessment in cyanotic infants after cardiovascular surgery[159]	Talwar S et al.	No specific newborn group
The effect of milrinone infusion on cerebral perfusion in neonates with congenital heart disease prior to cardiac surgery[160]	Bianchi M et al.	Lactate analyzed as outcome
Patent ductus arteriosus in preterm infants; experience of a tertiary referral neonatal intensive care unit: prevalence, complications, and management[161]	Soliman R et al.	Lactate as diagnosis of PDA
Impact of prenatal diagnosis of D transposition of the great arteries in the newborn who requires a balloon atrial septostomy[162]	Rosenthal J et al.	Lactate analyzed as outcome
Perinatal brain damage: predictive value of metabolic acidosis and the Apgar score.[163]	Ruth VJ et al.	Did not define lactate cut-off
Lactate as indicator for fetal and neonatal asphyxia.[164]	Dellenbach P et al.	Letter
Proposed entry criteria for postoperative cardiac extracorporeal membrane oxygenation after pediatric open heart surgery.[165]	Trittenwein G et al.	No specific newborn group
Gastrointestinal morbidity for the hybrid approach to hypoplastic left heart syndrome[166]	Luce W et al.	Abstract. Lactate analyzed as outcome
A composite outcome for neonatal cardiac surgery research[167]	Butts RJ et al.	Lactate as part of composite outcome
Serum lactates correlate with mortality after operations for complex congenital heart disease.[168]	Cheifetz IR et al.	No specific newborn group
Lactate and anion gap in asphyxiated neonates[169]	Oriot D et al.	Letter

An Elevated Low Cardiac Output Syndrome Score Is Associated With Morbidity in Infants After Congenital Heart Surgery[170]	Ulate K et al.	No specific newborn group. Lactate as part of a score
Correlations between near-infrared spectroscopy, perfusion index, and cardiac outputs in extremely preterm infants in the first 72 h of life[171]	Janaillac M et al.	Lactate correlated with NIRS and Echocardiography
Multicenter Validation of the Vasoactive-Ventilation-Renal Score as a Predictor of Prolonged Mechanical Ventilation After Neonatal Cardiac Surgery*[172]	Cashen K et al.	No outcome of interest
Predicting the likelihood of bronchopulmonary dysplasia in premature neonates[173]	Philpot PA et al.	Review
Early, mild fluid overload is associated with postoperative morbidity after neonatal cardiopulmonary bypass[174]	Udine M et al.	Authors did not compare lactate and outcomes
Predictors of Increased Lactate in Neonatal Cardiac Surgery: The Impact of Cardiopulmonary Bypass[175]	Nasr VG et al.	Lactate as outcome
Blood lactate concentrations and neonatal sepsis [87][176]	Molteni KH et al.	Letter
Serum anion gap in the differential diagnosis of metabolic acidosis in critically ill newborns[177]	Lorenz JM et al.	No outcome of interest
Early metabolic effects of sepsis in the preterm infant: Lactic acidosis and increased glucose requirement[178]	Fitzgerald MJ et al.	No information about lactate values
Secondary Increase of Lactate Levels in Asphyxiated Newborns during Hypothermia Treatment: Reflect of Suboptimal Hemodynamics (A Case Series and Review of the Literature)[179]	Balushi AA et al.	Case Series + Review
Multicenter validation of the vasoactiveventilation-renal score for neonatal cardiac surgery[180]	Mastropietro C et al.	Abstract. Not enough information.
[Evaluation on the early hemodynamic changes after cardiac surgery for congenital heart diseases in neonates].[181]	Qiu LS et al.	No newborn group
Potential clinical predictors of suspected early and late onset sepsis (EOS and LOS) in preterm newborns: A single tertiary center retrospective study[182]	Baizat M et al.	Lactate not analyzed
The relationship between B-type natriuretic peptide and echocardiographic and laboratory markers of circulatory status in preterm infants[183]	Konig K et al.	No outcome of interest. Lactate correlated to echocardiography
Myocardial ischaemia in neonates with perinatal asphyxia. Electrocardiographic, echocardiographic and enzymatic correlations.[184]	Barberi I et al.	Lactate not analyzed
Feeding associated neonatal necrotizing enterocolitis (Primary NEC) is an inflammatory bowel disease[185]	Clark DA et al.	Review
Patterns of lactate values after congenital heart surgery and timing of cardiopulmonary support.[186]	Hannan RL et al.	No specific newborn group

Prediction of progression of definite necrotising enterocolitis to need for surgery or death in preterm neonates.[187]	Srinivasjois R et al.	Inconsistent lactate values – wrong unit of measurement?
Prenatal diagnosis improves the postnatal cardiac function in a population-based cohort of infants with hypoplastic left heart syndrome[188]	Markkanen HK et al.	Lactate analyzed as outcome
Prediction of morbidity in SGA neonates: Are we using the right cord gas parameters to identify morbidity?[189]	Raghuraman N et al.	Lactate analyzed as outcome
Umbilical cord blood gas and lactate levels as a marker of birth asphyxia in neonates with particular reference to resource limited countries[190]	Waqar T et al.	Review
Prenatal diagnosis improves the perioperative condition of neonates requiring surgical intervention for coarctation but is associated with longer preoperative stay[191]	Houshmandi M et al.	Lactate analyzed as outcome
Cerebral tissue oxygenation index and lactate at 24 hours postoperative predict survival and neurodevelopmental outcome after neonatal cardiac surgery[192]	Aly SA et al.	Incomplete information about lactate (No standard deviation described). When lactate was analyzed in a multivariable regression, it was used with cTOI, not separately
Post-cardiotomy Rescue Extracorporeal Cardiopulmonary Resuscitation in Neonates with Single Ventricle After Intractable Cardiac Arrest: Attrition After Hospital Discharge and Predictors of Outcome.[193]	Polimenakos AC et al.	Very specific outcome: Extracorporeal cardiopulmonary resuscitation
Perioperative risk factors for impaired neurodevelopment after cardiac surgery in early infancy.[194]	Gunn JK et al.	Group analyzed < 2 months
Five-year neurocognitive and health outcomes after the neonatal arterial switch operation[195]	Neufeld RE et al.	No outcome of interest
Early Neurodevelopmental Outcomes in Children with Hypoplastic Left Heart Syndrome and Related Anomalies After Hybrid Procedure[196]	Khalid OM et al.	No outcome of interest
Intermediate-term outcomes of the arterial switch operation for transposition of great arteries in neonates: Alive but well?[197]	Freed DH et al.	No outcome of interest
Early childhood health, growth, and neurodevelopmental outcomes after complete repair of total anomalous pulmonary venous connection at 6 weeks or younger.[198]	Alton GY et al.	No outcome of interest
Optimal pulmonary to systemic blood flow ratio for best hemodynamic status and outcome early after Norwood operation.[199]	Photiadis J et al.	Population analyzed was the same as another included

		study from the same author
Lactate and intestinal fatty acid binding protein as essential biomarkers in neonates with necrotizing enterocolitis: ultrasonographic and surgical considerations[200]	Ahmed AEA et al.	No outcome of interest
Predictors and outcomes of early post-operative veno-arterial extracorporeal membrane oxygenation following infant cardiac surgery[201]	Kuraim GA et al.	No outcome of interest
Evaluation of urine output, lactate levels and lactate clearance in the transitional period in very low birth weight preterm infants[202]	Junior LKO et al.	Incomplete information – data test accuracy analysis not possible
Chronic Neuromotor Disability After Complex Cardiac Surgery in Early Life[203]	Ricci, MF et al.	No outcome of interest
The role of biochemical markers as early indicators of cardiac damage and prognostic parameters of perinatal asphyxia[204]	Simovic AM et al.	No outcome of interest

Table S3 – Assessment of risk of bias (Newcastle-Ottawa Scale)

	Newcastle-Ottawa Scale (Cohort studies)									
		Selection				Comparability		Outcome			Total
Author and Year	Study Design	Representativeness of exposed cohort	Selection of non-exposed cohort	Ascertainment of exposure	Outcome of interest	Study control for age	Study control for comorbidities	Assessment of outcome	Adequate duration of follow-up	Adequate follow-up of cohort	Total Score
Charpie JR 2000	PC	1	1	1	1	0	0	1	1	1	7
Polackova R 2017	PC	1	1	1	1	0	0	1	1	1	7
Lekhwani S 2010	RC	1	1	1	1	0	0	1	1	1	7
Tokuhisa T 2014	CC	1	1	1	1	0	0	1	1	1	7
Matsushita FY 2019	RC	1	1	1	1	0	0	1	1	1	7
Buijs EAB 2014	PC	0	1	1	1	0	0	1	1	1	6
Photiadis J 2006	PC	1	1	1	1	0	0	1	1	1	7
Amirnovin R 2013	PC	1	1	1	1	0	0	1	1	1	7
Li J 2012	RC	1	1	1	1	0	0	1	1	1	7
Shuhaiber J 2012	CC	1	1	1	1	1	1	1	1	1	9
Hayakawa M 2014	RC	1	1	1	1	0	0	1	1	1	7
Joffe AR 2007	PC	1	1	1	1	0	1	1	1	1	8
Manotas H 2017	RC	1	1	1	1	0	0	1	1	1	7
Liu X 2020	RC	1	1	1	1	1	1	1	1	1	9
Ouellete C 2019	RC	0	1	1	1	0	0	1	1	1	6
Miyamoto T 2008	RC	1	1	1	1	0	0	1	1	1	7
Rocha TS 2010	RC	1	1	1	1	0	0	1	1	1	7
Howard TS 2016	RC	1	1	1	1	1	1	1	1	1	9
Groenendaal F 2003	RC	1	1	1	1	0	0	1	1	1	7
Christmann M 2018	RC	1	1	1	1	0	0	1	1	1	7
Cheung PY 1994	RC	0	1	1	1	0	0	1	1	1	6
Phillips LA 2011	PC	1	1	1	1	1	1	1	1	1	9

Kessler U 2006	RC	0	1	1	1	0	0	1	1	1	6
Abubacker M 2002	RC	0	1	1	1	0	0	1	1	1	6
Verheijen PM 2010	RC	1	1	1	1	0	0	1	1	1	7
Araki S 2010	RC	1	1	1	1	0	0	1	1	1	7
Erdeve O 2019	PC	1	1	1	1	0	0	1	1	1	7
Chen D 2020	PC	1	1	1	1	1	1	1	1	1	9
Cheung PY 2002	PC	0	1	1	1	0	1	1	1	1	7
Cheung PY 2005	PC	1	1	1	1	0	0	1	1	1	7
Reppucci ML 2020	RC	0	1	1	1	0	0	1	1	1	6
Grayck EN 1995	RC	0	1	1	1	0	0	1	1	1	6
Fernandez HGC 2012	RC	1	1	1	0	1	0	1	1	1	7
Márquez-González H 2015	PC	1	1	1	1	1	1	1	1	1	9
Márquez-González-H 2015	PC	1	1	1	1	1	1	1	1	1	9
Murtuza B 2011	RC	1	1	1	1	0	0	1	1	1	7
Okur N 2018	PC	1	1	1	0	0	0	1	1	1	6
Tuten A 2017	PC	1	1	1	0	0	0	1	1	1	6
Deshpande SA 1996	PC	0	1	1	1	0	0	1	1	1	6
Chilinda GK 2018	PC	1	1	1	1	0	0	1	1	1	7
Haiju Z 2008	PC	1	1	1	1	0	0	1	1	1	7
Neacsu A 2020	RC	1	1	1	1	0	0	1	1	1	7
Mazouri A 2021	PC	0	1	1	0	0	0	1	1	1	5
Syed F 2019	PC	1	1	1	0	0	0	1	1	1	6
Karabayir N 2014	PC	1	1	1	1	0	0	1	1	1	7
Ozkiraz S 2013	CC	0	1	1	1	0	0	1	1	1	6
Simovic AM 2016	CC	1	1	1	1	0	0	1	1	1	7
Miletin J 2008	PC	1	1	1	1	0	0	1	1	1	7
Balushi AA 2017	RC	1	1	1	1	1	1	1	1	1	9

RC = Retrospective cohort; PC = Prospective cohort; CC = case control

References

83. Atallah, J.; Dinu, I.A.; Joffe, A.R.; Robertson, C.M.T.; Sauve, R.S.; Dyck, J.D.; Ross, D.B.; Rebeyka, I.M. Two-year survival and mental and psychomotor outcomes after the Norwood procedure: An analysis of the modified Blalock-Taussig shunt and right ventricle-to-pulmonary artery shunt surgical eras. *Circulation* **2008**, *118*, 1410–1418.
84. Bhat, P.; Hirsch, J.C.; Gelehrter, S.; Cooley, E.; Donohue, J.; King, K.; Gajarski, R.J. Outcomes of infants weighing three kilograms or less requiring extracorporeal membrane oxygenation after cardiac surgery. *Ann. Thorac. Surg.* **2013**, *95*, 656–661.
85. Sivarajan, V.; Penny, D.J.; Filan, P.; Brizard, C.; Shekerdemian, L.S. Impact of antenatal diagnosis of hypoplastic left heart syndrome on the clinical presentation and surgical outcomes: The Australian experience. *J. Paediatr. Child Health* **2009**, *45*, 112–117.
86. Gupta, P.; King, C.; Benjamin, L.; Goodhart, T.; Robertson, M.J.; Gossett, J.M.; Pesek, G.A.; DasGupta, R. Association of Hematocrit and Red Blood Cell Transfusion with Outcomes in Infants Undergoing Norwood Operation. *Pediatr. Cardiol.* **2015**, *36*, 1212–1218.
87. Topjian, A.A.; Clark, A.E.; Casper, T.C.; Berger, J.T.; Schleien, C.L.; Dean, J.M.; Moler, F.W. Early lactate elevations following resuscitation from pediatric cardiac arrest are associated with increased mortality. *Pediatr. Crit. Care Med.* **2013**, *14*, e380–e387.
88. Alves, R.L.; Aragão e Silva, A.L.; Kraychete, N.C.D.C.; Campos, G.O.; Martins, M.D.J.; Módolo, N.S.P. Intraoperative lactate levels and postoperative complications of pediatric cardiac surgery. *Paediatr. Anaesth.* **2012**, *22*, 812–817.
89. Burton, G.; Goot, B.; Da Cruz, E.; Kaufman, J. The use of arginine vasopressin in postoperative norwood patients. *Cardiol. Young* **2010**, *20*, S113.
90. Rossi, A.F.; Lopez, L.; Dobrolet, N.; Khan, D.; Bolivar, J. Hyperlactatemia in neonates admitted to the cardiac intensive care unit with critical heart disease. *Neonatology* **2010**, *98*, 212–216.
91. Kuzovlev, A.; Perepelitsa, S. Lactat acidosis—marker of severity of perinatal hypoxia. *Resuscitation* **2019**, *142*, e89–e90.
92. Boutaybi, N.; Steggerda, S.J.; Smits-Wintjens, V.E.H.J.; van Zwet, E.W.; Walther, F.J.; Lopriore, E. Early-onset thrombocytopenia in near-term and term infants with perinatal asphyxia. *Vox Sang.* **2014**, *106*, 361–367.
93. Kumar, N.; Yadav, A. Role of umbilical cord arterial pH and lactate in newborn assessment of term antenatal women with hypertensive disorders of pregnancy. *Clin. Epidemiol. Glob. Health* **2020**, *8*, 927–933.
94. De Azevedo, L.S.N.; Da Silva, A.N.; Oliveira, N.F.; Nogueira, P.C.K.; De Oliveira Iglesias, S.B.; Leite, H.P. Acute kidney injury assessed by prifle score and its relationship with metabolic markers and outcome. *Pediatr. Crit. Care Med.* **2012**, *13*, 620.
95. Ali Aydemir, N.; Harmandar, B.; Karaci, A.R.; Erdem, A.; Yurtseven, N.; Sasmazel, A.; Yekeler, I. Randomized comparison between mild and moderate hypothermic cardiopulmonary bypass for neonatal arterial switch operation. **2012**, *41*, 581–586.
96. Moustafa, A.A.; Antonios, M.A.M.; Abdellatif, E.M.; Hussain, A.H. Association of lactate/albumin ratio level to organ failure and mortality in severe sepsis in a pediatric intensive care unit in Egypt. *Turk. J. Pediatr.* **2018**, *60*, 691–701.
97. Doherty, D.R.; Parshuram, C.S.; Gaboury, I.; Hoskote, A.; Lacroix, J.; Tucci, M.; Joffe, A.; Choong, K.; Farrell, R.; Bohn, D.J.; et al. Hypothermia therapy after pediatric cardiac arrest. *Circulation* **2009**, *119*, 1492–1500.
98. Dogra, K.; Kaur, G.; Basu, S.; Chawla, D. Red Cell Transfusion Practices in Neonatal Intensive Care Unit: An Experience from Tertiary Care Centre. *Indian J. Hematol. Blood Transfus.* **2018**, *34*, 671–676.
99. Hickok, R.L.; Spaeder, M.C.; Berger, J.T.; Schuette, J.J.; Klugman, D. Postoperative Abdominal NIRS Values Predict Low Cardiac Output Syndrome in Neonates. *World J. Pediatr. Congenit. Hear. Surg.* **2016**, *7*, 180–184.
100. Hatherill, M.; McIntyre, A.G.; Wattie, M.; Murdoch, I.A. Early hyperlactataemia in critically ill children. *Intensive Care Med.* **2000**, *26*, 314–318.
101. Ergün, S.; Yildiz, O.; Güneş, M.; Akdeniz, H.S.; Öztürk, E.; Onan, İ.S.; Güzeltaş, A.; Haydin, S. Use of extracorporeal membrane oxygenation in postcardiotomy pediatric patients: Parameters affecting survival. *Perfusion* **2020**, *35*, 608–620.
102. Durward, A.; Tibby, S.M.; Skellett, S.; Austin, C.; Anderson, D.; Murdoch, I.A. The strong ion gap predicts mortality in children following cardiopulmonary bypass surgery. *Pediatr. Crit. Care Med.* **2005**, *6*, 281–285.
103. Scott, H.F.; Donoghue, A.J.; Gaieski, D.F.; Marchese, R.F.; Mistry, R.D. The utility of early lactate testing in undifferentiated pediatric systemic inflammatory response syndrome. *Acad. Emerg. Med.* **2012**, *19*, 1276–1280.
104. Sawyer, T.; Billimoria, Z.; Handley, S.; Smith, K.; Yalon, L.; Brogan, T.V.; Digeronimo, R. Therapeutic Plasma Exchange in Neonatal Septic Shock: A Retrospective Cohort Study. *Am. J. Perinatol.* **2020**, *37*, 962–969.
105. Nazir, M.; Wani, W.; Dar, S.A.; Mir, I.H.; Charoo, B.A.; Ahmad, Q.I.; Wajid, S. Lactate clearance prognosticates outcome in pediatric septic shock during first 24 h of intensive care unit admission. *J. Intensive Care Soc.* **2019**, *20*, 290–298.
106. Burkhardt, B.E.U.; Rücker, G.; Stiller, B. Prophylactic milrinone for the prevention of low cardiac output syndrome and mortality in children undergoing surgery for congenital heart disease. *Cochrane Database Syst. Rev.* **2015**, *2015*. doi: 10.1002/14651858.CD009515.pub2
107. García-Hernández, J.A.; Benítez-Gómez, I.L.; Martínez-López, A.I.; Praena-Fernández, J.M.; Cano-Franco, J.; Loscertales-Abril, M. Prognostic markers of mortality after congenital heart defect surgery. *An. Pediatr. (Barc).* **2012**, *77*, 366–373.
108. Mackie, A.S.; Alton, G.Y.; Dinu, I.A.; Joffe, A.R.; Roth, S.J.; Newburger, J.W.; Robertson, C.M. Clinical outcome score predicts the need for neurodevelopmental intervention after infant heart surgery. *J. Thorac. Cardiovasc. Surg.* **2013**, *145*, 1248–1254.e2.
109. Cashen, K.; Reeder, R.; Dalton, H.J.; Berg, R.A.; Shanley, T.P.; Newth, C.J.; Pollack, M.M.; Wessel, D.; Carcillo, J.; Harrison, R.; et al. Functional Status of Neonatal and Pediatric Patients After Extracorporeal Membrane Oxygenation. *Pediatr. Crit. Care Med.* **2017**, *18*, 561–570.
110. Molina Hazan, V.; Gonen, Y.; Vardi, A.; Keidan, I.; Mishali, D.; Rubinshtein, M.; Yakov, Y.; Paret, G. Blood lactate levels differ significantly between surviving and nonsurviving patients within the same risk-adjusted Classification for Congenital Heart Surgery (RACHS-1) group after pediatric cardiac surgery. *Pediatr. Cardiol.* **2010**, *31*, 952–960.

111. Neamtu, M.L.; Dobrota, L. Lactic acidosis: A highly indicator of unfavorable outcome in critically ill children. *Intensive Care Med.* **2013**, *39*, S176–S177.
112. Vari, D.; Behere, S.; Spurrier, E.; Baffa, J. Low-dose prostaglandin e1 for congenital heart disease: Is it time to revisit the dosing guidelines. *J. Am. Coll. Cardiol.* **2019**, *73*, 600.
113. Botha, P.; Deshpande, S.R.; Wolf, M.; Heard, M.; Alsoufi, B.; Kogon, B.; Kanter, K. Extracorporeal membrane oxygenator support in infants with systemic-pulmonary shunts. *J. Thorac. Cardiovasc. Surg.* **2016**, *152*, 912–918.
114. Polimenakos, A.C.; Wojtyla, P.; Smith, P.J.; Rizzo, V.; Nater, M.; El Zein, C.F.; Ilbawi, M.N. Post-cardiotomy extracorporeal cardiopulmonary resuscitation in neonates with complex single ventricle: Analysis of outcomes. *Eur. J. Cardiothorac. Surg.* **2011**, *40*, 1396–1405; discussion 1405.
115. Rhodes, L.A.; Erwin, W.C.; Borasino, S.; Cleveland, D.C.; Alten, J.A. Central Venous to Arterial Co2 Difference after Cardiac Surgery in Infants and Neonates*. *Pediatr. Crit. Care Med.* **2017**, *18*, 228–233.
116. Castro-Rodríguez, C.O.; Rodríguez-Hernández, L.; de Jesús Estrada-Loza, M.; Herrera-Márquez, J.R.; Gómez-Salvador, M.; Flores-Lujano, J.; & Núñez-Enríquez, J.C. Prognostic factors associated with postoperative morbidity in children with isolated ventricular septal defect. *Rev. Med. Inst. Mex. Seguro Soc.* **2015**, *53*, S324–S335.
117. Killinger, J.S.; Hsu, D.T.; Schleien, C.L.; Mosca, R.S.; Hardart, G.E. Children undergoing heart transplant are at increased risk for postoperative vasodilatory shock. *Pediatr. Crit. Care Med.* **2009**, *10*, 335–340.
118. Kalyanaraman, M.; DeCampli, W.M.; Campbell, A.I.; Bhalala, U.; Harmon, T.G.; Sandiford, P.; McMahon, C.K.; Shore, S.; Yeh, T.S. Serial blood lactate levels as a predictor of mortality in children after cardiopulmonary bypass surgery. *Pediatr. Crit. Care Med.* **2008**, *9*, 285–288.
119. Schumacher, K.R.; Reichel, R.A.; Vlasic, J.R.; Yu, S.; Donohue, J.; Gajarski, R.J.; Charpie, J.R. Rate of increase in serum lactate level risk-stratifies infants after surgery for congenital heart disease. *J. Thorac. Cardiovasc. Surg.* **2014**, *148*, 589–595.
120. Kubicki, R.; Grohmann, J.; Siepe, M.; Benk, C.; Humburger, F.; Rensing-Ehl, A.; Stiller, B. Early prediction of capillary leak syndrome in infants after cardiopulmonary bypass. *Eur. J. Cardiothorac. Surg.* **2013**, *44*, 275–281.
121. Kramer, P.; Mommsen, A.; Miera, O.; Photiadis, J.; Berger, F.; Schmitt, K.R.L. Survival and Mid-Term Neurologic Outcome after Extracorporeal Cardiopulmonary Resuscitation in Children. *Pediatr. Crit. Care Med.* **2020**, *21*, e316–e324.
122. Mildh, L.H.; Pettilä, V.; Sairanen, H.I.; Rautiainen, P.H. Cardiac troponin T levels for risk stratification in pediatric open heart surgery. *Ann. Thorac. Surg.* **2006**, *82*, 1643–1648.
123. Kanaris, C.; Ramanathan, G.; Pritchard, L.; Stibbards, S. Risk stratification of critically ill children and neonates with acute general surgical pathology requiring stabilisation, transfer to tertiary care facility and factors predicting mortality. *Pediatr. Crit. Care Med.* **2018**, *19*, 91.
124. PR, A.-S.; Lazo-Cárdenas, C.; Rodríguez-Hernández, L.; Márquez-González, H.; JA, G.-S. [Mortality-associated factors in pediatric patients with Blalock-Taussig shunt]. *Rev. Med. Inst. Mex. Seguro Soc.* **2014**, *52*, S62–S67.
125. Olshove, V.; Berndsen, N.; Nawathe, P.; Robert, S.; Phillips, A. Acute kidney injury scoring system is a better predictor of increased length compared to inotrope score. *Cardiol. Young* **2017**, *27*, S344.
126. Manso, P.; Ferreira, M.; Silva, T.; Turquetto, A.; Caneo, L.; Santos, J.; Amato, L.; Carmona, F. Risk factors for mechanical ventilation time after congenital heart surgery. *Cardiol. Young* **2017**, *27*, S348–S349.
127. Rossi, A.F.; Khan, D.M.; Hannan, R.; Bolivar, J.; Zaidenweber, M.; Burke, R. Goal-directed medical therapy and point-of-care testing improve outcomes after congenital heart surgery. *Intensive Care Med.* **2005**, *31*, 98–104.
128. Dodge-Khatami, J.; Gottschalk, U.; Eulenburg, C.; Wendt, U.; Schnegg, C.; Rebel, M.; Reichenspurner, H.; Dodge-Khatami, A. Prognostic value of perioperative near-infrared spectroscopy during neonatal and infant congenital heart surgery for adverse in-hospital clinical events. *World J. Pediatr. Congenit. Heart Surg.* **2012**, *3*, 221–228.
129. Siegel, L.B.; Dalton, H.J.; Hertzog, J.H.; Hopkins, R.A.; Hannan, R.L.; Hauser, G.J. Initial postoperative serum lactate levels predict survival in children after open heart surgery. *Intensive Care Med.* **1996**, *22*, 1418–1423.
130. Rastan, A.J.; Walther, T.; Alam, N.A.; Daehnert, I.; Borger, M.A.; Mohr, F.W.; Janousek, J.; Kostelka, M. Moderate versus deep hypothermia for the arterial switch operation--experience with 100 consecutive patients. *Eur. J. Cardiothorac. Surg.* **2008**, *33*, 619–625.
131. Ricci, Z.; Garisto, C.; Favia, I.; Vitale, V.; Di Chiara, L.; Cogo, P.E. Levosimendan infusion in newborns after corrective surgery for congenital heart disease: Randomized controlled trial. **2012**, *38*, 1198–1204.
132. Scherer, B.; Moser, E.A.S.; Brown, J.W.; Rodefeld, M.D.; Turrentine, M.W.; Mastropietro, C.W. Vasoactive-ventilation-renal score reliably predicts hospital length of stay after surgery for congenital heart disease. *J. Thorac. Cardiovasc. Surg.* **2016**, *152*, 1423–1429.e1.
133. Brix, N.; Sellmer, A.; Jensen, M.S.; Pedersen, L.V.; Henriksen, T.B. Predictors for an unsuccessful INtubation-SURfactant-Extubation procedure: A cohort study. *BMC Pediatr.* **2014**, *14*, 155.
134. Solé, A.; Jordan, I.; Bobillo, S.; Moreno, J.; Balaguer, M.; Hernández-Platero, L.; Segura, S.; Cambra, F.J.; Esteban, E.; Rodríguez-Fanjul, J. Venoarterial extracorporeal membrane oxygenation support for neonatal and pediatric refractory septic shock: More than 15 years of learning. *Eur. J. Pediatr.* **2018**, *177*, 1191–1200.
135. Schlapbach, L.J.; MacLaren, G.; Festa, M.; Alexander, J.; Erickson, S.; Beca, J.; Slater, A.; Schibler, A.; Pilcher, D.; Millar, J.; et al. Prediction of pediatric sepsis mortality within 1 h of intensive care admission. *Intensive Care Med.* **2017**, *43*, 1085–1096.
136. Munoz, R.; Laussen, P.C.; Palacio, G.; Zienko, L.; Piercey, G.; Wessel, D.L. Changes in whole blood lactate levels during cardiopulmonary bypass for surgery for congenital cardiac disease: An early indicator of morbidity and mortality. *J. Thorac. Cardiovasc. Surg.* **2000**, *119*, 155–162.
137. Nagata, H.; Glick, L.; Lougheed, J.; Grattan, M.; Mondal, T.; Thakur, V.; Schwartz, S.M.; Jaeggi, E. Prenatal Diagnosis of Transposition of the Great Arteries Reduces Postnatal Mortality: A Population-Based Study. *Can. J. Cardiol.* **2020**, *36*, 1592–1597.

138. Woods, P.; Halliday, R.; Skowno, J. Utilisation of near infrared spectroscopy (NIRS) in monitoring haemodynamic stability of infants with hypoplastic left heart syndrome (HLHS) in the presurgical setting. *J. Paediatr. Child Health* **2013**, *49*, 68–69.
139. Garisto, C.; Favia, I.; Ricci, Z.; Chiara, L.D.; Morelli, S.; Giorni, C.; Vitale, V.; Picardo, S.; Di Donato, R.M. Initial single-center experience with levosimendan infusion for perioperative management of univentricular heart with ductal-dependent systemic circulation. *World J. Pediatr. Congenit. Heart Surg.* **2010**, *1*, 292–299.
140. Jaeggi, E.; Glick, L.; Lougheed, J.; Mondal, T.; Rosenberg, H.; Thakur, V.; Schwartz, S.; Nagata, H. Prenatal detection of transposition of the great arteries does not reduce mortality and morbidity. *Cardiol. Young* **2016**, *26*, S33.
141. Rodríguez-Fanjul, J.; Solé, A.; Bobillo, S.; Moreno, J.; Segura, S.; Esteban, E.; Balaguer, M.; Jordan, I. Extracorporeal membrane oxygenation for refractory septic shock in children: Our institution's results. *Eur. J. Heart Fail.* **2017**, *19*, 23–24.
142. Morris, K.P.; McShane, P.; Stickley, J.; Parslow, R.C. The relationship between blood lactate concentration, the Paediatric Index of Mortality 2 (PIM2) and mortality in paediatric intensive care. *Intensive Care Med.* **2012**, *38*, 2042–2046.
143. Boigner, H.; Brannath, W.; Hermon, M.; Stoll, E.; Burda, G.; Trittenwein, G.; Golej, J. Predictors of mortality at initiation of peritoneal dialysis in children after cardiac surgery. *Ann. Thorac. Surg.* **2004**, *77*, 61–65.
144. Weber, R.W.; Stiasny, B.; Ruecker, B.; Fasnacht, M.; Cavigelli-Brunner, A.; Valsangiacomo Buechel, E.R. Prenatal Diagnosis of Single Ventricle Physiology Impacts on Cardiac Morbidity and Mortality. *Pediatr. Cardiol.* **2019**, *40*, 61–70.
145. Amini, S.; Abbaspour, H.; Morovatdar, N.; Robabi, H.N.; Soltani, G.; Tashnizi, M.A. Risk factors and outcome of acute kidney injury after congenital heart surgery: A prospective observational study. *Indian J. Crit. Care Med.* **2017**, *21*, 847–851.
146. Siddiqui, I.; Jafri, L.; Abbas, Q.; Raheem, A.; Haque, A.U. Relationship of serum procalcitonin, c-reactive protein, and lactic acid to organ failure and outcome in critically ill pediatric population. *Indian J. Crit. Care Med.* **2018**, *22*, 91–95.
147. Nishibe, S.; Tsujita, M. The impact of intraoperative vasopressin infusion in complex neonatal cardiac surgery. *Interact. Cardiovasc. Thorac. Surg.* **2012**, *15*, 966–972.
148. Ressia, L.; Calevo, M.G.; Lerzo, F.; Carleo, A.M.; Petrucci, L.; Montobbio, G. Beneficial effect of fenoldopam mesylate in preventing peak blood lactate level during cardiopulmonary bypass for paediatric cardiac surgery. *Interact. Cardiovasc. Thorac. Surg.* **2014**, *19*, 178–182.
149. Garcia Guerra, G.; Joffe, A.R.; Senthilselvan, A.; Kutsogiannis, D.J.; Parshuram, C.S.. Incidence of milrinone blood levels outside the therapeutic range and their relevance in children after cardiac surgery for congenital heart disease. *Intensive Care Med.* **2013**, *39*, 951–957.
150. Örmeci, T.; Alkan-Bozkaya, T.; Özyüksel, A.; Ersoy, C.; Ündar, A.; Akçevin, A.; Türkoğlu, H. Correlation between cerebral-renal near-infrared spectroscopy and ipsilateral renal perfusion parameters as clinical outcome predictors after open heart surgery in neonates and infants. *Artif. Organs* **2015**, *39*, 53–58.
151. Davidson, J.; Tong, S.; Hancock, H.; Hauck, A.; Da Cruz, E.; Kaufman, J. Prospective validation of the vasoactive-inotropic score and correlation to short-term outcomes in neonates and infants after cardiothoracic surgery. *Intensive Care Med.* **2012**, *38*, 1184–1190.
152. Huang, S.C.; Wu, E.T.; Chen, Y.S.; Chang, C.I.; Chiu, S.; Chi, N.H.; Wu, M.H.; Wang, S.S.; Lin, F.Y.; Ko, W.J. Experience with extracorporeal life support in pediatric patients after cardiac surgery. *ASAIO J.* **2005**, *51*, 517–521.
153. Kinoshita, M.; Hawkes, C.P.; Ryan, C.A.; Dempsey, E.M. Perfusion index in the very preterm infant. *Acta Paediatr.* **2013**, *102*, e398–e401.
154. Budniok, T.; ElSayed, Y.; Louis, D. Effect of Vasopressin on Systemic and Pulmonary Hemodynamics in Neonates. *Am. J. Perinatol.* **2020**, *38*, 1330–1334.
155. Sawada, M.; Ueda, K.; Matsuo, K.; Tokumasu, S.; Ogino, K.; Hayashi, T.; Saito, M.; Kubota, M.; Takahashi, A.; Watabe, S.; et al. Continuous renal replacement therapy in the NICU.; Ten years' experience in a singlecenter. *Pediatr. Nephrol.* **2015**, *30*, 2235.
156. Pugni, L.; Ronchi, A.; Bizzarri, B.; Consonni, D.; Pietrasanta, C.; Ghirardi, B.; Fumagalli, M.; Ghirardello, S.; Mosca, F. Exchange transfusion in the treatment of neonatal septic shock: A ten-year experience in a neonatal intensive care unit. *Int. J. Mol. Sci.* **2016**, *17*, 695.
157. Algra, S.O.; Kornmann, V.N.N.; Van Der Tweel, I.; Schouten, A.N.J.; Jansen, N.J.G.; Haas, F. Increasing duration of circulatory arrest, but not antegrade cerebral perfusion, prolongs postoperative recovery after neonatal cardiac surgery. *J. Thorac. Cardiovasc. Surg.* **2012**, *143*, 375–382.
158. Hoffman, T.M.; Wernovsky, G.; Atz, A.M.; Kulik, T.J.; Nelson, D.P.; Chang, A.C.; Bailey, J.M.; Akbary, A.; Kocsis, J.F.; Kaczmarek, R.; et al. Efficacy and safety of milrinone in preventing low cardiac output syndrome in infants and children after corrective surgery for congenital heart disease. *Circulation* **2003**, *107*, 996–1002.
159. Talwar, S.; Bansal, A.; Sahu, M.K.; Singh, S.P.; Choudhary, S.K.; Airan, B. Vasoactive inotropic score and outcome assessment in cyanotic infants after cardiovascular surgery. *J. Card. Crit. Care* **2018**, *2*, 25–31.
160. Bianchi, M.O.; Cheung, P.Y.; Phillipos, E.; Aranha-Netto, A.; Joynt, C. The effect of milrinone infusion on cerebral perfusion in neonates with congenital heart disease prior to cardiac surgery. *Arch. Dis. Child.* **2012**, *97*, A93–A94.
161. Soliman, R.M.; Mostafa, F.A.; Abdelmassih, A.; Sultan, E.; Mosallam, D. Patent ductus arteriosus in preterm infants; experience of a tertiary referral neonatal intensive care unit: Prevalence, complications, and management. *Egypt. Pediatr. Assoc. Gaz.* **2020**, *68*, 1–9.
162. Rosenthal, J.; Ravi, P.; Eckersly, L.; Houshmandi, M.; Savard, W.; Hornberger, L. Impact of prenatal diagnosis of D transposition of the great arteries in the newborn who requires a balloon atrial septostomy. *Cardiol. Young* **2017**, *27*, S320–S321.
163. Ruth, V.J.; Raivio, K.O. Perinatal brain damage: Predictive value of metabolic acidosis and the Apgar score. *BMJ* **1988**, *297*, 24–27.
164. Dellenbach, P.; Haberey, P. Lactate as indicator for fetal and neonatal asphyxia. *Lancet (Lond. Engl.)* **1982**, *1*, 907.
165. Trittenwein, G.; Pansi, H.; Graf, B.; Golej, J.; Burda, G.; Hermon, M.; Marx, M.; Wollenek, G.; Trittenwein, H.; Pollak, A. Proposed entry criteria for postoperative cardiac extracorporeal membrane oxygenation after pediatric open heart surgery. *Artif. Organs* **1999**, *23*, 1010–1014.

166. Luce, W.; Schwartz, R.; Beauseau, W.; Giannone, P.; Hashiguchi, B.; Cheatham, J.P.; Galantowicz, M.; Cua, C.L. Gastrointestinal morbidity for the hybrid approach to hypoplastic left heart syndrome. *Cardiol. Young* **2009**, *19*, 147.
167. Butts, R.J.; Scheurer, M.A.; Zyblewski, S.C.; Wahlquist, A.E.; Nietert, P.J.; Bradley, S.M.; Atz, A.M.; Graham, E.M. A composite outcome for neonatal cardiac surgery research. *J. Thorac. Cardiovasc. Surg.* **2014**, *147*, 428–433.
168. Cheifetz, I.M.; Kern, F.H.; Schulman, S.R.; Greeley, W.J.; Ungerleider, R.M.; Meliones, J.N. Serum lactates correlate with mortality after operations for complex congenital heart disease. *Ann. Thorac. Surg.* **1997**, *64*, 735–738.
169. Oriot, D.; Nasimi, A.; Berthier, M.; Marlin, S.; Hubert, A.; Follet-Bouhamed, C. Lactate and anion gap in asphyxiated neonates [5]. *Arch. Dis. Child. Fetal Neonat. Ed.* **1998**, *78*, F80.
170. Ulate, K.P.; Yanay, O.; Jeffries, H.; Baden, H.; Di Gennaro, J.L.; Zimmerman, J. An Elevated Low Cardiac Output Syndrome Score Is Associated With Morbidity in Infants After Congenital Heart Surgery. *Pediatr. Crit. Care Med.* **2017**, *18*, 26–33.
171. Janaillac, M.; Beausoleil, T.P.; Barrington, K.J.; Raboisson, M.-J.; Karam, O.; Dehaes, M.; Lapointe, A. Correlations between near-infrared spectroscopy, perfusion index, and cardiac outputs in extremely preterm infants in the first 72 h of life. *Eur. J. Pediatr.* **2018**, *177*, 541–550.
172. Cashen, K.; Costello, J.M.; Grimaldi, L.M.; Gowda, K.M.N.; Moser, E.A.S.; Piggott, K.D.; Wilhelm, M.; Mastropietro, C.W. Multicenter Validation of the Vasoactive-Ventilation-Renal Score as a Predictor of Prolonged Mechanical Ventilation After Neonatal Cardiac Surgery*. *Pediatr. Crit. Care Med.* **2018**, *19*, 1015–1023.
173. Philpot, P.A.; Bhandari, V. Predicting the likelihood of bronchopulmonary dysplasia in premature neonates. *Expert Rev. Respir. Med.* **2019**, *13*, 871–884.
174. Udine, M.; Borasino, S.; Alten, J.; Kirklin, J.; McNeal, S.; Xie, R.; Naftel, D.; Hock, K.; Dabal, R.; Cleveland, D. Early, mild fluid overload is associated with postoperative morbidity after neonatal cardiopulmonary bypass. *World J. Pediatr. Congenit. Heart Surg.* **2018**, *9*, NP24.
175. Nasr, V.G.; Staffa, S.J.; Boyle, S.; Regan, W.; Brown, M.; Smith-Parrish, M.; Kaza, A.; DiNardo, J.A. Predictors of Increased Lactate in Neonatal Cardiac Surgery: The Impact of Cardiopulmonary Bypass. *J. Cardiothorac. Vasc. Anesth.* **2020**, *35*, 148–153.
176. Molteni, K.H.; Fitzgerald, M.; Goto, M.; Myers, T.F.; Zeller, W.P. Blood lactate concentrations and neonatal sepsis [5]. *J. Pediatr.* **1993**, *123*, 493–494.
177. Lorenz, J.M.; Kleinman, L.I.; Markarian, K.; Oliver, M.; Fernandez, J. Serum anion gap in the differential diagnosis of metabolic acidosis in critically ill newborns. *J. Pediatr.* **1999**, *135*, 751–755.
178. Fitzgerald, M.J.; Goto, M.; Myers, T.F.; Zeller, W.P. Early metabolic effects of sepsis in the preterm infant: Lactic acidosis and increased glucose requirement. *J. PEDIATR.* **1992**, *121*, 951–955.
179. Al Balushi, A.; Guilbault, M.-P.; Wintermark, P. Secondary Increase of Lactate Levels in Asphyxiated Newborns during Hypothermia Treatment: Reflect of Suboptimal Hemodynamics (A Case Series and Review of the Literature). *AJP Rep.* **2015**, *6*, e48–e58.
180. Mastropietro, C.; Cashen, K.; Narayana, K.M.; Piggott, G.K.; Wilhelm, M.; Costello, J. Multicenter validation of the vasoactiveventilation-renal score for neonatal cardiac surgery. *Crit. Care Med.* **2016**, *44*, 108.
181. Qiu, L.S.; Liu, J.F.; Zhu, L.M.; Xu, Z.M. Evaluation on the early hemodynamic changes after cardiac surgery for congenital heart diseases in neonates. *Zhonghua Er Ke Za Zhi = Chin. J. Pediatr.* **2009**, *47*, 662–666.
182. Baizat, M.; Zaharie, G.; Iancu, M.; Muresan, D.; Hășmășanu, M.; Procopciuc, L.M. Potential clinical predictors of suspected early and late onset sepsis (EOS and LOS) in preterm newborns: A single tertiary center retrospective study. *Clin. Lab.* **2019**, *65*, 1299–1308.
183. König, K.; Drew, S.; Walsh, G.; Burke, E.; Barfield, C.; Watkins, A.; Collins, C. The relationship between B-type natriuretic peptide and echocardiographic and laboratory markers of circulatory status in preterm infants. *Monatsschr. Kinderheilkd.* **2011**, *159*, 98.
184. Barberi, I.; Calabrò, M.P.; Cordaro, S.; Gitto, E.; Sottile, A.; Prudente, D.; Bertuccio, G.; Consolo, S. Myocardial ischaemia in neonates with perinatal asphyxia. Electrocardiographic, echocardiographic and enzymatic correlations. *Eur. J. Pediatr.* **1999**, *158*, 742–747.
185. Clark, D.A.; Munshi, U.K. Feeding associated neonatal necrotizing enterocolitis (Primary NEC) is an inflammatory bowel disease. *Pathophysiology* **2014**, *21*, 29–34.
186. Hannan, R.L.; Ybarra, M.A.; White, J.A.; Ojito, J.W.; Rossi, A.F.; Burke, R.P. Patterns of lactate values after congenital heart surgery and timing of cardiopulmonary support. *Ann. Thorac. Surg.* **2005**, *80*, 1464–1468.
187. Srinivasjois, R.; Nathan, E.; Doherty, D.; Patole, S. Prediction of progression of definite necrotising enterocolitis to need for surgery or death in preterm neonates. *J. Matern. Fetal. Neonatal Med.* **2010**, *23*, 695–700.
188. Markkanen, H.K.; Pihkala, J.I.; Salminen, J.T.; Saarinen, M.M.; Hornberger, L.K.; Ojala, T.H. Prenatal diagnosis improves the postnatal cardiac function in a population-based cohort of infants with hypoplastic left heart syndrome. *J. Am. Soc. Echocardiogr.* **2013**, *26*, 1073–1079.
189. Raghuraman, N.; Tuuli, M.G.; Macones, G.A.; Cahill, A.G.; Stout, M.J. Prediction of morbidity in SGA neonates: Are we using the right cord gas parameters to identify morbidity? *Am. J. Obs. Gynecol.* **2018**, *218*, S304.
190. Waqar, T.; Haque, K.N. Umbilical cord blood gas and lactate levels as a marker of birth asphyxia in neonates with particular reference to resource limited countries. *Pak. Paediatr. J.* **2013**, *37*, 197–203.
191. Houshmandi, M.; Eckersley, L.; Savard, W.; Fruitman, D.; Mills, L.; Hornberger, L. Prenatal diagnosis improves the perioperative condition of neonates requiring surgical intervention for coarctation but is associated with longer preoperative stay. *Can. J. Cardiol.* **2017**, *33*, S40.
192. Aly, S.A.; Zurakowski, D.; Glass, P.; Skurow-Todd, K.; Jonas, R.A.; Donofrio, M.T. Cerebral tissue oxygenation index and lactate at 24 hours postoperative predict survival and neurodevelopmental outcome after neonatal cardiac surgery. *Congenit. Heart Dis.* **2017**, *12*, 188–195.

193. Polimenakos, A.C.; Rizzo, V.; El-Zein, C.F.; Ilbawi, M.N. Post-cardiotomy Rescue Extracorporeal Cardiopulmonary Resuscitation in Neonates with Single Ventricle After Intractable Cardiac Arrest: Attrition After Hospital Discharge and Predictors of Outcome. *Pediatr. Cardiol.* **2017**, *38*, 314–323.
194. Gunn, J.K.; Beca, J.; Hunt, R.W.; Goldsworthy, M.; Brizard, C.P.; Finucane, K.; Donath, S.; Shekerdemian, L.S. Perioperative risk factors for impaired neurodevelopment after cardiac surgery in early infancy. *Arch. Dis. Child.* **2016**, *101*, 1010–1016.
195. Neufeld, R.E.; Clark, B.G.; Robertson, C.M.; Moddemann, D.M.; Dinu, I.A.; Joffe, A.R.; Sauve, R.S.; Creighton, D.E.; Zwaigenbaum, L.; Ross, D.B.; et al. Five-year neurocognitive and health outcomes after the neonatal arterial switch operation. *J. Thorac. Cardiovasc. Surg.* **2008**, *136*, 1413–1421, 1421.e1-1421.e2.
196. Khalid, O.M.; Harrison, T.M. Early Neurodevelopmental Outcomes in Children with Hypoplastic Left Heart Syndrome and Related Anomalies After Hybrid Procedure. *Pediatr. Cardiol.* **2019**, *40*, 1591–1598.
197. Freed, D.H.; Robertson, C.M.T.; Sauve, R.S.; Joffe, A.R.; Rebeyka, I.M.; Ross, D.B.; Dyck, J.D. Intermediate-term outcomes of the arterial switch operation for transposition of great arteries in neonates: Alive but well? *J. Thorac. Cardiovasc. Surg.* **2006**, *132*, 845–852.e2.
198. Alton, G.Y.; Robertson, C.M.; Sauve, R.; Divekar, A.; Nettel-Aguirre, A.; Selzer, S.; Joffe, A.R.; Rebeyka, I.M.; Ross, D.B. Early childhood health, growth, and neurodevelopmental outcomes after complete repair of total anomalous pulmonary venous connection at 6 weeks or younger. *J. Thorac. Cardiovasc. Surg.* **2007**, *133*, 905–911.
199. Photiadis, J.; Sinzobahamvya, N.; Fink, C.; Schneider, M.; Schindler, E.; Brecher, A.M.; Urban, A.E.; Asfour, B. Optimal pulmonary to systemic blood flow ratio for best hemodynamic status and outcome early after Norwood operation. *Eur. J. Cardiothorac. Surg.* **2006**, *29*, 551–556.
200. El-Abd Ahmed, A.; Hassan, M.H.; Abo-Halawa, N.; Abdel-Razik, G.M.; Moubarak, F.A.; Sakhr, H.M. Lactate and intestinal fatty acid binding protein as essential biomarkers in neonates with necrotizing enterocolitis: Ultrasonographic and surgical considerations. *Pediatr. Neonatol.* **2020**, *61*, 481–489.
201. Kuraim, G.A.; Garros, D.; Ryerson, L.; Moradi, F.; Dinu, I.A.; Garcia Guerra, G.; Moddemann, D.; Bond, G.Y.; Robertson, C.M.T.; Joffe, A.R. Predictors and outcomes of early post-operative veno-arterial extracorporeal membrane oxygenation following infant cardiac surgery. *J. Intensive Care* **2018**, *6*, 1–12.
202. Junior, L.K.O.; Carmona, F.; Aragon, D.C.; Gonçalves-Ferri, W.A. Evaluation of urine output, lactate levels and lactate clearance in the transitional period in very low birth weight preterm infants. *Eur. J. Pediatr.* **2021**, *180*, 91–97.
203. Ricci, M.F.; Andersen, J.C.; Joffe, A.R.; Watt, M.J.; Moez, E.K.; Dinu, I.A.; Guerra, G.G.; Ross, D.B.; Rebeyka, I.M.; Robertson, C.M.T. Chronic Neuromotor Disability After Complex Cardiac Surgery in Early Life. *Pediatrics* **2015**, *136*, e922–e933.
204. Simović, A.M.; Košutić, J.L.; Prijić, S.M.; Knežević, J.B.; Vujić, A.J.; Stojanović, N.D. The role of biochemical markers as early indicators of cardiac damage and prognostic parameters of perinatal asphyxia. *Uloga Biohemijskih Markera Kao Ranih Indik. Oštećenja Srca I Progn. Parametara Perinat. Asfiksije* **2014**, *71*, 149–155.

Disclaimer/Publisher's Note: The statements, opinions and data contained in all publications are solely those of the individual author(s) and contributor(s) and not of MDPI and/or the editor(s). MDPI and/or the editor(s) disclaim responsibility for any injury to people or property resulting from any ideas, methods, instructions or products referred to in the content.
